# Partial indoor residual spraying with pirimiphos-methyl as an effective and cost-saving measure for the control of *Anopheles gambiae* s.l. in northern Ghana

**DOI:** 10.1038/s41598-021-97138-1

**Published:** 2021-09-10

**Authors:** Sylvester Coleman, Yemane Yihdego, Ellie Sherrard-Smith, Churcher S. Thomas, Dereje Dengela, Richard M. Oxborough, Samuel K. Dadzie, Daniel Boakye, Frank Gyamfi, Kwasi Obiri-Danso, Ben Johns, Lilly V. Siems, Bradford Lucas, Jon Eric Tongren, Sixte Zigirumugabe, Dominic Dery, Christen Fornadel, Kristen George, Allison Belemvire, Jenny Carlson, Seth R. Irish, Jennifer S. Armistead, Aklilu Seyoum

**Affiliations:** 1 PMI VectorLink Project, Abt Associates, Plot 11 Waterson Road, Fuo, Tamale, Ghana; 2 PMI VectorLink Project, Abt Associates, 6130 Executive Blvd, Rockville, MD 20852 USA; 3 Department of Infectious Disease Epidemiology, MRC Centre for Global Infectious Disease Analysis, Imperial College London, London, UK; 4 Noguchi Memorial Institute for Medical Research, University of Ghana, Legon, Ghana; 5 Department of Theoretical and Applied Biology, Kwame Nkrumah University of Science and Technology, Kumasi, Ghana; 6 US. President’s Malaria Initiative, U.S. Agency for International Development, Accra, Ghana; 7 U.S. President’s Malaria Initiative, U.S. Agency for International Development, Washington, DC USA; 8 U.S. President’s Malaria Initiative, Centers for Disease Control and Prevention, Atlanta, Georgia USA

**Keywords:** Entomology, Malaria

## Abstract

The scale up of indoor residual spraying (IRS) and insecticide treated nets have contributed significantly to global reductions in malaria prevalence over the last two decades. However, widespread pyrethroid resistance has necessitated the use of new and more expensive insecticides for IRS. Partial IRS with pirimiphos-methyl in experimental huts and houses in a village-wide trial was evaluated against *Anopheles gambiae* s.l. in northern Ghana. Four different scenarios in which either only the top or bottom half of the walls of experimental huts were sprayed, with or without also spraying the ceiling were compared. Mortality of *An. gambiae* s.l. on partially sprayed walls was compared with the standard procedures in which all walls and ceiling surfaces are sprayed. A small-scale trial was then conducted to assess the effectiveness, feasibility, and cost of spraying only the upper walls and ceiling as compared to full IRS and no spraying in northern Ghana. Human landing catches were conducted to estimate entomological indices and determine the effectiveness of partial IRS. An established transmission dynamics model was parameterized by an analysis of the experimental hut data and used to predict the epidemiological impact and cost effectiveness of partial IRS for malaria control in northern Ghana. In the experimental huts, partial IRS of the top (IRR 0.89, *p* = 0.13) or bottom (IRR 0.90, *p* = 0.15) half of walls and the ceiling was not significantly less effective than full IRS in terms of mosquito mortality. In the village trial, the annual entomological inoculation rate was higher for the unsprayed control (217 infective bites/person/year (ib/p/yr)) compared with the fully and partially sprayed sites, with 28 and 38 ib/p/yr, respectively. The transmission model predicts that the efficacy of partial IRS against all-age prevalence of malaria after six months would be broadly equivalent to a full IRS campaign in which 40% reduction is expected relative to no spray campaign. At scale, partial IRS in northern Ghana would have resulted in a 33% cost savings ($496,426) that would enable spraying of 36,000 additional rooms. These findings suggest that partial IRS is an effective, feasible, and cost saving approach to IRS that could be adopted to sustain and expand implementation of this key malaria control intervention.

## Introduction

Indoor residual spraying (IRS) of insecticides, insecticide treated nets (ITNs) and artemisinin combination therapies have contributed to the substantial reduction of the malaria burden in the last two decades^1^. However, IRS programs face challenges in sustaining these gains due to emerging resistance to insecticides, the high cost of non-pyrethroid insecticides, increasing operational costs, and limited overall financial resources available for malaria vector control. The use of insecticides for malaria vector control was dominated globally by organochlorine (DDT) and pyrethroid insecticides between 2000 and 2009^2^. However, increased utilization of pyrethroid insecticides both for IRS and insecticide treated nets (ITNs) for malaria vector control and agricultural use has led to widespread pyrethroid resistance in malaria vectors across most of malaria endemic sub-Saharan Africa^3–5^. According to the 2020 World Health Organization (WHO) World Malaria Report, globally a total of 73 countries confirmed resistance to at least one insecticide in one malaria vector species between 2010 and 2019^6^.

The shift from relatively cheaper pyrethroid insecticides, first to carbamates and organophosphates, and more recently to neonicotinoid-based insecticides^7^ has led to reductions in the geographic coverage of IRS, primarily due to the necessary shift towards more expensive insecticides^6^. In 2010, when pyrethroid insecticides were primarily used, 5% of the global population at risk of malaria was protected by IRS, but this declined to 3% in 2018^8^ and 2% in 2019^6^. In 2009, of 15 countries that conducted IRS with support from the U.S. President’s Malaria Initiative (PMI), 13 sprayed pyrethroid insecticides; this decreased to 7 of 16 countries in 2013. During the same period the number of countries that sprayed carbamates (bendiocarb) increased from 1 of 15 in 2009 to 9 of 16 in 2013^9^**.** After the development of a long-lasting organophosphate insecticide, pirimiphos-methyl CS, there was a significant shift away from the use of pyrethroid and carbamate insecticides for IRS, with all 11 PMI-supported IRS program countries spraying pirimiphos-methyl CS in 2016^10^. More recently, neonicotinoid (clothianidin-based) insecticides have become available and are being used for IRS in 13 sub-Saharan African countries with support from PMI.

The current cost of a pyrethroid sachet (K-Othrine WG25) in 2020, $1.18 (all costs are in US dollars), is considerably less than the initial ($23.50) and current ($16.19) price per bottle of pirimiphos-methyl CS, both with an equivalent quantity of insecticide to spray 250 m^2^ at the WHO recommended target dosages. The current cost of recently WHO prequalified IRS formulations that contain clothianidin, SumiShield (WG 50%, Sumitomo) and Fludora Fusion (WP-SB, Bayer), is $14.50 per sachet.

In this context, identification of innovative cost-saving approaches for implementation of IRS will be necessary to sustain or even increase the gains of this critical malaria intervention. The current practice for IRS recommended by WHO is for all interior wall and ceiling surfaces in a house where vectors might come into contact with the insecticide to be uniformly sprayed^11^. An alternative approach is to reduce the surface area sprayed by only spraying part of the walls with or without also spraying the ceilings, potentially reducing the quantity of insecticide needed as well as operational effort and expenses, resulting in substantial cost savings. Furthermore, spraying only the upper part of the wall and ceiling may not require moving furniture outside of houses, which could substantially save household preparation time and increase acceptance by the community especially where removing household material is particularly difficult.

While other partial IRS approaches for malaria vector control have been trialed in Mexico against *An. albimanus*^12^, *An. flavirostris* in the Philippines^13^ and *An. sacharovi* in Lebanon^14^ with promising results, none of these alternative approaches to IRS have been widely adopted. Here, we aimed to leverage the extensive experience, best practices and efficiencies established through PMI-supported IRS programs to identify a sustainable and scalable alternative spraying approach for IRS that is equally impactful and cost-effective as standard IRS as it is currently deployed. In the initial assessment of a partial IRS approach described here, mortality of the main local mosquito vector, *Anopheles gambiae* s.l., was measured in experimental huts in which only the bottom or top half of walls, with or without spraying the ceiling, were sprayed as compared to positive control huts in which walls and ceilings were fully sprayed with pirimiphos-methyl CS. This was followed with a small-scale, village-level trial in eight communities in northern Ghana where the impact of partial spraying of upper walls and ceilings with pirimiphos-methyl CS on the human biting rate (HBR), parity and the annual entomological inoculation rate (EIR) of *An. gambiae* s.l. were evaluated. The top half of the walls and ceilings was selected based on comparable level of efficacy to full IRS in the experimental huts and the possibility of eliminating the need to remove furniture from houses while spraying. Cost savings and household acceptability of this approach were also assessed to determine the feasibility of scaling up partial IRS for malaria vector control. Finally, an established transmission model^15–17^, parameterized by the experimental hut data, was used to predict the epidemiological impact and cost effectiveness of partial IRS for malaria control in northern Ghana.

## Methods

### Study sites

**Figure 1:**
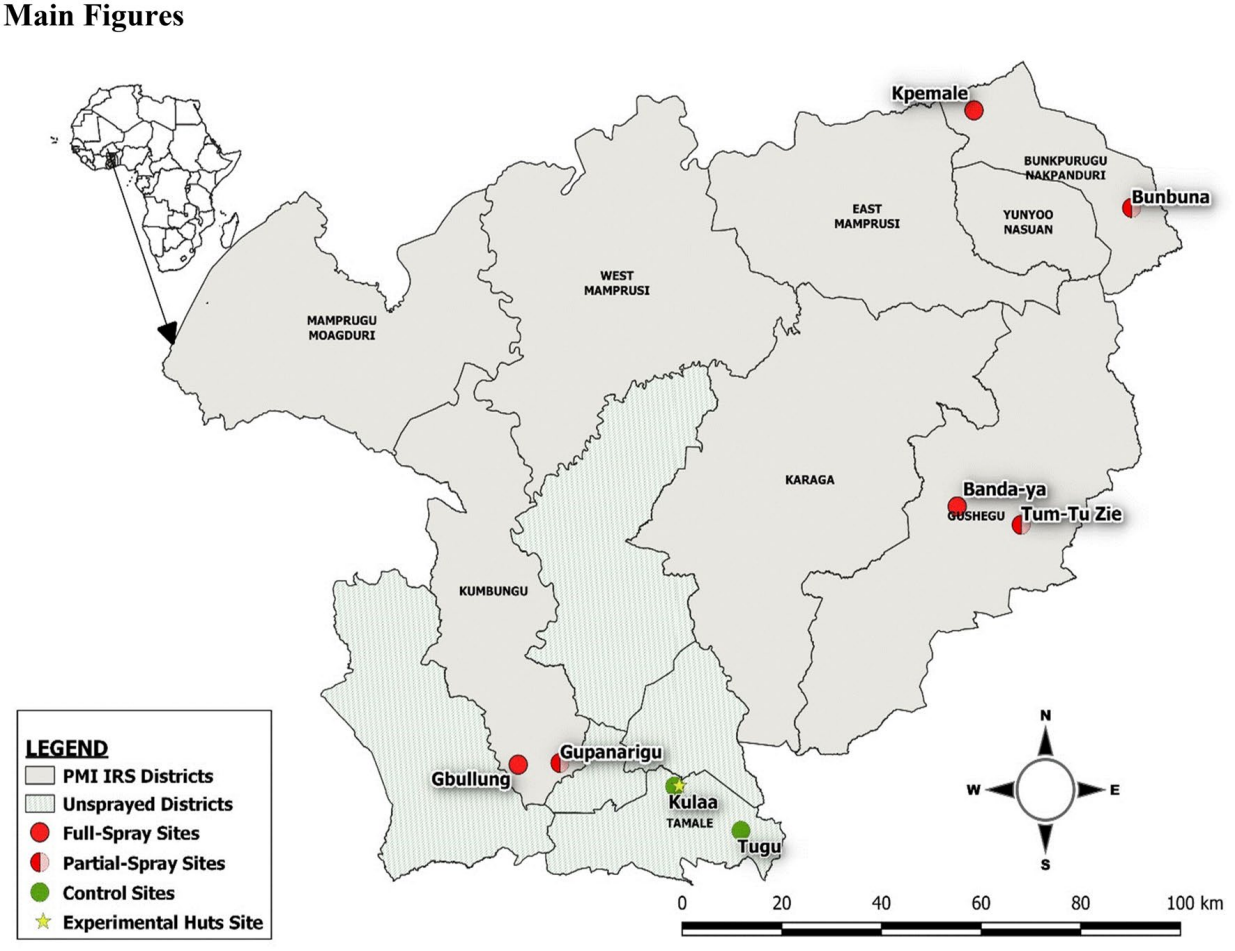
Partial IRS Study Sites in northern Ghana. Partial IRS was evaluated in an experimental hut study at one site (yellow star) in Tamale Metropolitan District and subsequently in a village-scale trial conducted in four districts in northern Ghana. In Kumbungu, Bunkpurugu Nakpanduri, and Gushegu districts one community was partially sprayed (red/pink semi-circles) and one was fully sprayed (red circles). The trial also included two unsprayed communities (green circles) in Tamale Metropolitan District.

An experimental hut trial was conducted from May to November 2018 at Kulaa, a community located approximately 12 km from Tamale in northern Ghana (Fig. 1). IRS has not previously been implemented in this community and insecticide susceptibility tests using WHO susceptibility tests showed that the local vector populations were susceptible to pirimiphos-methyl^18^.

A village-scale trial was conducted in May 2019 in six sprayed communities, with one partially sprayed and one fully sprayed village in three districts in northern Ghana (Northern and North East regions) (Fig. 1): Gbullung and Gupanarigu (Kumbungu District (KUD)); Kpemale and Bunbuna (Bunkpurugu Nakpanduri District (BND)); and Banda-ya and Tum-Tu Zie (Gushegu District (GUD)). The history of IRS and insecticides used in these districts is presented in Supplementary Table S1 (Supplementary file 1). Two communities (Kulaa and Tugu) in Tamale metropolis (TML) with no history of IRS were used as control sites.

All sites are rural and located in Ghana’s northern savannah zone, with very similar ecological characteristics, and where *An. gambiae* s.l. is the major malaria vector. *An. gambiae* s.s. has previously been found to be the predominant species (90%) but co-exists with *An. coluzzii* (9%) and *An. arabiensis* (1%) in this area^19^. The typical housing style in the study communities are predominantly round huts with mud walls and conical thatched roofs, though rectangular houses with metal roofs are also found in the area (Supplementary Fig. S1). The interior walls of the huts are mostly plastered with either mud or cement. A 7–8 months residual activity of pirimiphos-methyl CS has been previously reported for mud and cement surfaces in the study area^18^. Insecticide treated nets have been distributed widely throughout northern Ghana, and therefore ITN ownership was expected to be similar across all IRS and non-IRS districts in this study^20^. Prior to the start of the study (June 2017) the percentage of the population in the Northern region of Ghana with access to an ITN was 77%, while usage was estimated at 51%^20^.

### Experimental hut study

#### Experimental huts

Twelve experimental huts (Supplementary Fig. S2) were constructed based on the West Africa experimental hut design described in WHO guidelines for the evaluation of ITNs and IRS^21^. Each hut was made from concrete bricks with a corrugated iron roof and a ceiling with thick polyethylene tarpaulin on the interior surface. The total sprayable surface area of the huts was similar, ranging from 27.37 to 28.64 m^2^. Each hut stood on a concrete base surrounded by a water-filled moat to prevent entry of ants that would otherwise carry off dead mosquitoes. Entry of mosquitoes occurred via four modified slits that were 3 cm wide, located on three sides of the hut. Mosquitoes were able to egress into a small veranda projecting from the fourth side^22^. The floor was covered with white plastic material to ease the collection of knocked down mosquitoes in the morning.

#### Experimental hut study design

Two huts were randomly assigned to each of the different treatment and control scenarios as follows: (1) all walls and ceiling sprayed to serve as positive controls (fully sprayed), (2) sprayed with only water to serve as unsprayed negative controls, (3) bottom half of walls and ceiling sprayed, (4) only bottom half of walls sprayed, (5) top half of walls and ceiling sprayed, and (6) only top half of walls sprayed. The walls of the huts were marked with chalk at the middle section of the window (about 1.3 m from the floor) on each side of the room and lines were drawn to partition the room into upper and lower parts. The experimental huts were then sprayed with pirimiphos-methyl CS at 1 g/m^2^ following standard procedures on July 13, 2018, using a trained and experienced spray operator from the PMI VectorLink project in Ghana.

Twelve volunteer sleepers recruited from the community were rotated among the huts each night, to minimize any bias that could arise from differences in individual attractiveness to mosquitoes. One sleeper entered each hut at 8:00 pm local time and remained inside until 6:00 am, with a supervisor ensuring that individuals rotated and slept in different experimental huts each night. Each sleeper slept in each hut for one night during a complete rotation of 12 nights, under unholed and untreated mosquito nets.

Mosquitoes were collected pre- and post- spray in each hut between 6:00 am and 8:00 am each morning by trained field technicians using mouth aspirators. Pre-spray mosquito collections were carried out daily for two rounds (between June 19 and July 12, 2018) each with 12 nights to determine baseline mosquito density in each hut and to record resting positions on the wall (top half and bottom half) both inside of the hut and on the veranda, as well as on ceilings. Mosquitoes resting on the windows and untreated nets were also collected and recorded during the morning mosquito collections from the huts. Mosquitoes were scored by location as dead or alive and as blood fed or unfed.

Post-spray mosquito collections were carried out for nine rounds (between July 16 and November 30, 2018) each for 12 consecutive nights. Dead mosquitoes were collected from the floor of the huts and resting mosquitoes were collected from the walls, ceiling, and verandas each morning using mouth aspirators. Mosquitoes were scored by location as dead or alive and as blood fed or unfed. Live mosquitoes were placed in small holding cups and provided with access to sugar solution to assess mortality after 24 h. Efficacy was expressed in terms of percentage mortality of free-flying, wild *An. gambiae* s.l. both at the time of collection (immediate) and after a 24-h holding period, referred to as total mortality in the results section. All *Anophele*s mosquitoes were identified to species morphologically^23^ and a sub-sample of the *An. gambiae* s.l. were further identified to the sibling species level with polymerase chain reaction (PCR)^24,25^. Ten dead mosquitoes from the insectary were placed in a petri dish and left on the floor of each hut to check that predation by ants was not affecting the data. This was to prevent errors in under estimating mortality, the key indicator in the experimental hut study.

### Village-scale trial

#### Trial design

In each of the three IRS districts, one community received partial IRS while the other received full IRS with pirimiphos-methyl CS (Fig. 1). The pair of communities in each district were at least 2–3 km from each other to minimize possible impacts from adjacent communities. The following treatments were compared in the village-scale trial: (1) full IRS in which all walls and ceiling of sleeping rooms were sprayed (positive controls) in Banda-ya, Gbullung and Kpemale communities and (2) partial IRS in which the top half of walls and ceiling of sleeping rooms were sprayed in Bunbuna, Tum-Tu Zie and Gupanarigu. Two additional communities, Kulaa and Tugu, (unsprayed sites) served as negative controls for the study.

#### IRS implementation

Spraying was conducted between May 16 and May 26, 2019 across all communities by trained spray operators (SOPs) and supervised by trained spray supervisors under the PMI VectorLink Ghana project. All SOPs were trained together, however SOPs that sprayed the partial IRS communities were additionally taken through field simulations of partial IRS to enable them to adjust their technique accordingly. The wall surfaces of eligible rooms as well as ceilings (if present) were sprayed with pirimiphos-methyl CS at a rate of 1 g/m^2^. Ceilings of both grass thatched houses and those with metal roofs were sprayed in the same way. A team of markers (four people) preceded the spray teams in the partial IRS communities to mark the rooms into upper and lower halves. The different sections of the walls of each room were measured and equally divided into two halves, typically close to the base of the windows (if present). The spray coverage in both partial and full IRS sites was calculated as the total number of rooms sprayed out of the total found at the respective communities. Cone wall bioassays were conducted to confirm spray quality and assess the residual life of the insecticides, using standard WHO protocol^26^.

Based on historical data on SOP output in northern Ghana, the mean daily spray target set for each SOP was 18 rooms per day for full IRS communities with the average size of sprayable room estimated as 41.8 m^2^. Assuming that only about two thirds of the total surface area per room would be sprayed and less household preparation time anticipated, SOP daily output is expected to increase by one third (about 6–7 rooms more) for a day’s work. The estimated daily output expected in partial IRS communities was 25 rooms per SOP per day.

#### Entomological data collection

Pre-spray (March–May 2019) and post-spray (June-December 2019) indoor and outdoor human landing catches (HLCs) were conducted monthly at three different houses per night for four nights, for a total of twelve houses per month in each of the partial IRS, full IRS, and unsprayed communities. The same houses were used for sampling mosquitoes each month. The human biting rate (HBR) reported as bites/person/night (monthly) calculated by indoor vs outdoor, were estimated from the HLCs.

All *Anopheles* mosquitoes were identified to species morphologically^23^. About 50–60 (per site per month) of unfed *An. gambiae* s.l. from HLCs were dissected to assess the number parous by observing the degree of coiling in the ovarian tracheoles^27^. Parity rates were estimated from the dissections, as the number of parous female mosquitoes/total number of female mosquitoes dissected.

A total of 7495 *An. gambiae* s.l. (about 18%) of mosquitoes collected by HLC were randomly selected and examined for sporozoite infection by enzyme-linked immunosorbent assay (ELISA)^28^, with similar proportions analyzed for partial, full, and unsprayed communities. Of this number 640 (about 9%) *An. gambiae* s.l. were randomly selected and further identified to sibling species level by PCR^24,25^. The number of infectious bites per person per unit time (monthly) were estimated by multiplying the sporozoite rates with the human biting rates. Annual EIR was estimated as the sum of monthly EIRs in the year (March –December). January and February are the driest months of the year in northern Ghana and mosquito collections are typically very low and therefore 0 infective bites per person were assumed for the two months. The human biting rates, parity and entomological inoculation rates of the predominant vector were estimated and compared between treatments.

Spray quality and residual efficacy of insecticide were determined using the WHO cone wall bioassay with insecticide-susceptible *An. gambiae* Kisumu strain. The cone bioassays were performed on three main types of sprayed surfaces: mud walls (in traditional houses), cement walls (in modern houses), and wood, used for doors and windows.

Both spray quality and residual efficacy were estimated from the percentage mortality of the exposed mosquitoes from the WHO cone bioassays on the different types of sprayed surfaces. The fumigant effect of the sprayed insecticide on *An. gambiae* Kisumu strain was also assessed at the time of the assays, using wire cages mounted 10 cm from the sprayed wall surfaces.

#### Estimation of cost savings

On the day of spraying, note takers recorded the total amount of time that each SOP spent spraying a room, total number of households an SOP sprayed in a day, amount of insecticide used per day, total amount of insecticide used to spray each community, and average number of rooms sprayed per bottle of insecticide. The amount of time spent to spray a house was determined by recording the start and end time of spraying in both partial and full IRS communities. Note takers were instructed to start the timer once they heard the SOP start spraying and to stop the timer when the SOP opened the door to exit the room. This information was then translated into associated costs. Time spent to remove the household furniture was not included to estimate additional cost savings. Members of the communities have the practice of removing household furniture during the previous spray campaigns, therefore when the spray operators arrived for spraying, furniture were already removed from the houses.

#### IRS acceptability surveys

Qualitative surveys were conducted seven months post-spray (in December 2019) in a subset of households in the IRS communities to determine the acceptability of partial IRS as compared with full IRS. Based on the assumption that all 720 households, defined as a collection of structures considered as one unit (often referred to as a compound) with an adult male or female head and a unique identifier, that were reported across the six IRS communities were sprayed, 188 were randomly selected for the survey. This equated to approximately 30% of households from each treatment area (full IRS and partial IRS). Using household listings from the communities, the survey team visited identified households. Verbal consent was sought from each head of the household before an interview was conducted. Only persons above 18 years of age were interviewed.

### Statistical analyses

#### Experimental hut study

The number of mosquitoes entering the huts and the proportion of mosquitoes that were dead relative to the positive controls were compared by treatment scenario. The outcome of interest was the cumulative number of dead mosquitoes at 24 h, as a proportion of the total number of mosquitoes captured (mortality rate). Only *An. gambiae* s.l. (female) mosquitoes were considered in the analyses. Negative binomial models were used to assess differences in mortality rates across treatment groups. The number of mosquitoes captured was used as the exposure variable, where the number of dead mosquitoes at 24 h was the outcome of interest. The treatment categories were included in the model as a fixed effect, with fully sprayed huts used as the comparator. Thus, the model tested if the mortality rate across treatments was different from a fully sprayed hut. In addition, the hut number, the volunteer sleeper, and time since spray (in two-week periods) were included as random effects.

#### Village-scale trial

The indoor and outdoor HBR for each treatment was calculated as the number of mosquitoes caught by HLC in a night divided by the number of people catching mosquitoes. In all study sites, two people collected mosquitoes indoors and two people collected outdoors (four people in total who rotated between indoors and outdoors hourly throughout the night) meaning that the time of exposure to mosquito bites was the same for all observations. Thus, linear hierarchical regression was used to calculate average differences between partial and full IRS sites and not negative binomial regression because, with constant exposure, the results take on the properties of a continuous variable, and linear regression has been shown to have fewer Type I errors than negative binomial regression^29^. In the linear hierarchical regression, type of treatment was included as the main outcome of interest, month of observation as a fixed effect, and community, household, and place of collection (indoor/outdoor) as random effects. Robust standard errors were used to account for any non-normality in the error term (due to, for example, truncation of the error term at zero bites). The same method was used to assess differences in the percentage of dissected mosquitoes found to be parous.

### Modeling analysis for predicting the impact and cost effectiveness of IRS

#### Entomological impact

The experimental hut data collated in 2018 in Ghana were used to estimate the variable impact that partial IRS of walls and ceilings inside houses may have on entomological indices measured. Briefly, time-dependent logistic binomial functions were fit to the mortality and deterrence data observed in the experimental hut trials following Sherrard-Smith et al.^15^. The experimental hut trial in northern Ghana provided volunteers with untreated and unholed nets so that the blood-feeding estimates observed represent a situation with both untreated nets and spraying. Therefore, we assumed blood-feeding and exiting estimates (which are affected by proportions of mosquitoes successfully blood-feeding) from a systematic review of experimental hut trials with pirimiphos-methyl CS^15^ and combined these with estimates of measurable entomological outcomes (mortality and deterrence) from this experimental hut study to parameterize the transmission model for malaria and then used the model to determine the potential public health impact of partial IRS.

#### Epidemiological impact

The transmission model tracks transmission of *Plasmodium falciparum* between human hosts and *Anopheles* mosquito vectors. The differential equations and associated assumptions of the original transmission model^17^ have been comprehensively reported in the Supplementary Material from Griffin et al.^16^, Walker et al.^30^ and Winskill et al.^31^. Unless otherwise noted, parameter estimates use those presented in these papers. The code is publically available here: https://github.com/jamiegriffin/Malaria_simulation. The model has been extensively fitted to data on the relationship between vector density, EIR, parasite prevalence, uncomplicated malaria, severe disease and death^16,17,32–34^. Details of the data used to inform the model and parameter estimates derived from these data are provided in Supplementary File 2.

A generic scenario that broadly reflects the Northern region of Ghana was used to explore the potential lost protective efficacy (measured as the cases averted per 1,000 people per year, and the relative reduction in prevalence after 2, 4, 6 and 8 months post-spraying) that is provided by the respective spray campaign strategy that occurs when IRS is administered to the different sections of the wall or ceilings measured in the experimental huts. Ghana is generally a perennial transmission setting but in the northern savannah zone, there is a peak in transmission in June to October^35^, hence the spray campaign takes place in May, at the optimal time relative to this transmission peak.

Simplifying assumptions were made, centered on a range of pyrethroid resistance levels in the local malaria vector, net use, as well as coverage and possible impact of seasonal malaria chemoprevention in the region. Using these assumptions, inferences were made from the modelling exercise on the altered impact of IRS when partially sprayed. Given the high coverage of rooms sprayed in the village trial we simulate that 90% of people are protected by the IRS application (IRS application was simulated to take place simultaneously on May 16^th^, 2019). Different effectiveness of the IRS intervention was then driven by the efficacies estimated using the experimental hut data for the respective spray scenarios (lower walls, upper walls, lower walls and ceilings, upper walls and ceilings, or full spraying). We assumed no resistance to pirimiphos-methyl, the active ingredient in Actellic 300 CS^36^. The main assumptions used to make these inferences are described in detail in Supplementary File 2.

#### Comparison of effectiveness with full and partial IRS

The transmission model simulations provided scenarios to compare the potential public health impact of partially IRS with pirimiphos-methyl CS. We simulated the all-age clinical cases per 1000 people per year for 1-year following the spray campaigns for each of the simulation scenarios. We also provided a comparison of prevalence at 2, 4, 6 and 8-months after spraying. These metrics provide measures to compare effectiveness between a full IRS and various partial IRS scenarios relative to no IRS campaign. To do this, we use the efficacy equation:

$$\% Efficacy=100 \times \frac{\left(C-T\right)}{C}$$

where *C* is the counterfactual trial arm simulated, without any IRS deployed, and *T* is the trial arm with some level of IRS implemented—be it partial or fully sprayed.

These estimates are used to generate cost-effectiveness estimates for the application of partial IRS where only upper walls and ceilings are covered. To do this, we estimate the all-age clinical cases averted per strategy (partial: upper walls and ceiling; full: full spray) per person per year following the IRS campaign. We multiply this by the cost per person for the partial or full IRS strategy tested in the trial to give the cost per case averted.

### Ethical permission

Ethical approvals for this study were obtained from the Institutional Review Board (IRB) of Noguchi Memorial Institute for Medical Research and Abt Associates Ethics Committee before starting the study. Informed consent was obtained from all volunteers (sleepers) and mosquito collectors that participated in the study as well as from community members interviewed on IRS acceptability surveys.

All methods were performed in accordance with relevant guidelines and regulations.

## Results

### Experimental hut study

A total of 550 *An. gambiae* s.l. mosquitoes were caught from all 12 experimental huts over 24 collection nights during the pre-spray baseline period. All mosquitoes were alive at the time of collection. The average density of *An. gambiae* s.l. at baseline was 1.9 per hut per day (1.7–2.2, 95% CI). Of the mosquitoes that entered and remained inside the experimental huts, ceilings were the preferred resting locations (44%), though a small proportion were collected from the walls (7%). Of those that exited the huts, the top part of the veranda (22% of total collected) was the preferred resting location (Supplementary Fig. S3). In all instances, all 10 dead mosquitoes from the insectary placed in petri dishes in the huts were retrieved the next morning ruling out any predation by ants.

A total of 6317 *An. gambiae* s.l. mosquitoes were caught in the experimental huts during the 108 nights of post-spray collections. Of those mosquitoes from which DNA was amplified (n = 674) and processed with PCR, 89.2% were identified as *An. gambiae*, 4.7% as *An. coluzzii*, 4.0% as *An. arabiensis* and 2% as hybrids of *An. gambiae* and *An. coluzzii*. During the nine rounds of post-spray mosquito collections, 76.8% of *An. gambiae* s.l. captured in the fully sprayed huts were dead at 24 h (Supplementary Table S2). Total mortality of *An. gambiae* s.l. in the huts over the duration of post spraying data collection with the lower wall and ceiling sprayed was 68.2%, while in the huts with the upper wall and ceiling sprayed it was 66.5%. Huts with only the upper wall or lower wall sprayed had overall mortality rates of 40.9% and 45.7%, respectively. The percentage of mosquitoes fed were similar in all treatment and control huts, ranging from 25.5% in the fully sprayed huts to 33.5% in unsprayed huts (Supplementary Table S2).

**Figure 2:**
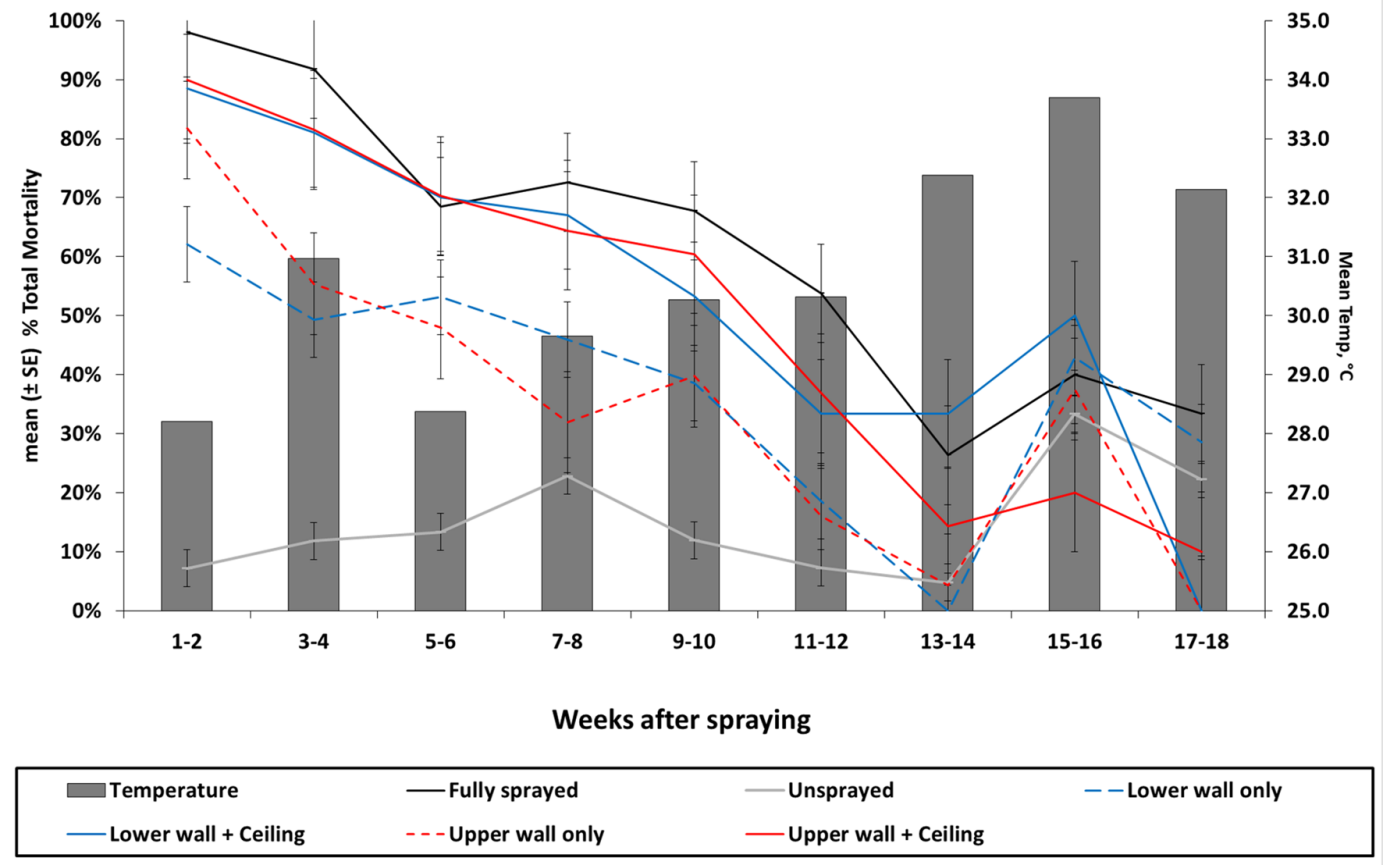
*Anopheles gambiae* s.l. mean total mortality in experimental huts with different IRS scenarios up to 18 weeks post-intervention. Post-spray mosquito mortality decreased gradually for fully and partially sprayed treatment scenarios and the rate of decrease was consistent over time. Mosquito mortality in experimental huts where upper wall + ceiling (solid red line) or lower wall + ceiling (solid blue line) was not significantly different to that observed in fully sprayed huts (solid black line), whereas spraying only the upper walls (dashed red line) or lower walls (dashed blue line) resulted in significantly lower mosquito mortality. Mosquito mortality in untreated huts (solid gray line) was consistently low throughout the study. A spike in mortality was observed across all treatments in weeks 15–16, during which the mean weekly temperature (gray bars) in experimental huts shown also increased.

Total mortality decreased gradually for each treatment scenario and the rate of decrease was consistent over time (Fig. 2). Mortality in untreated control huts was generally low, with a mean of 14.5% throughout the 18-week period. However, mortality in untreated huts was particularly high during weeks 15 and 16, at 32.0%. A similar spike in mortality was also observed across most treatment scenarios during this period, the timing of which coincided with a brief increase in mean daily temperature. In adjusted negative binomial regression (when including random effects for time since spray, volunteer sleepers, and treatment huts), neither the huts with the lower wall and ceiling sprayed (IRR 0.90, *p* = 0.15) or the huts with the upper wall and ceiling sprayed (IRR 0.89, *p* = 0.13) were inferior to the fully sprayed huts (Supplementary Table S3). All other treatments (unsprayed control, lower wall only, and upper wall only) were significantly inferior to the fully sprayed huts.

#### Predicting entomological outcomes from experimental hut data

**Figure 3:**
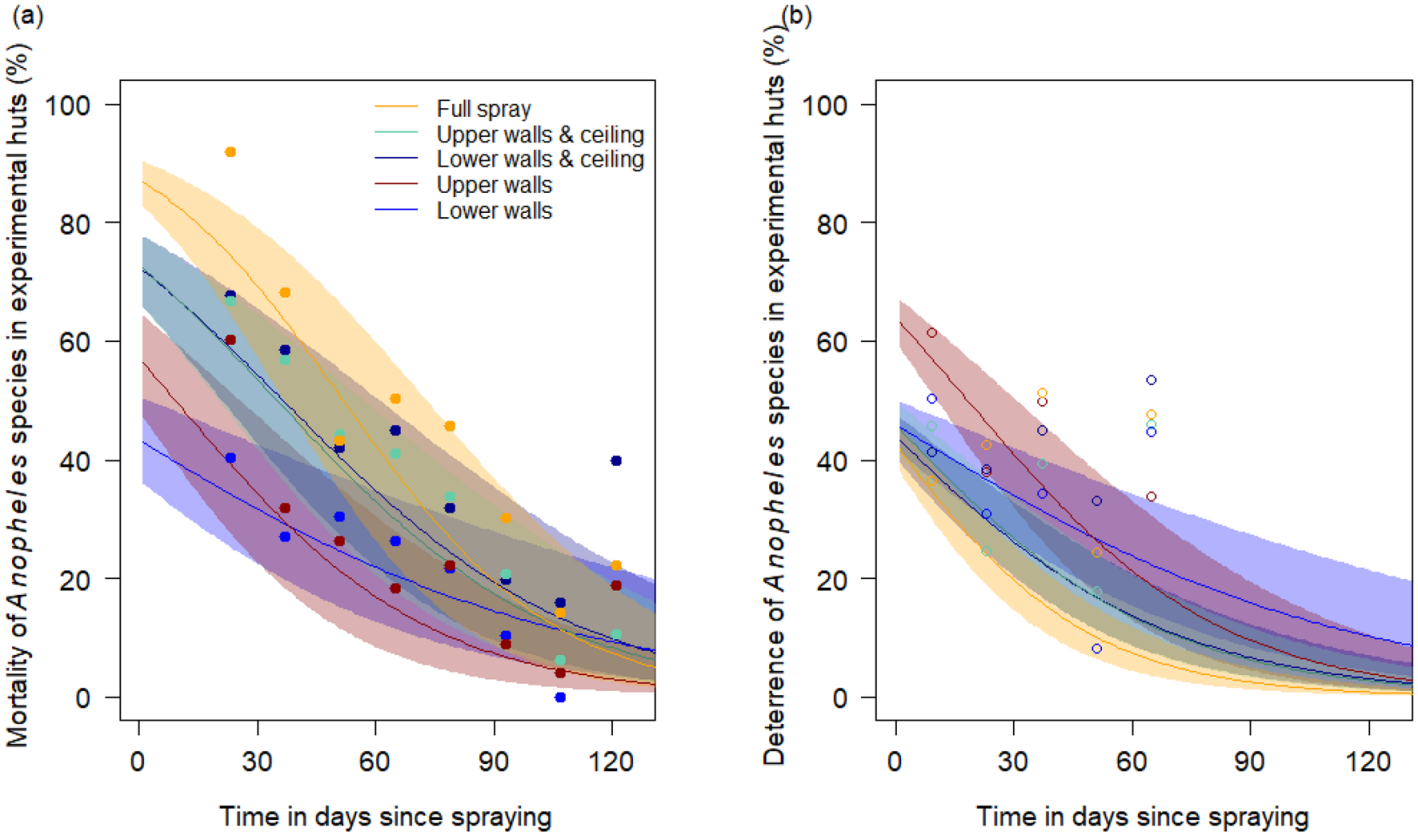
Predicted outcomes of *Anopheles* mosquito feeding attempts following full or partial IRS with pirimiphos-methyl CS. (**a**) A logistic binomial function was fit to the mosquito mortality measured in experimental hut trials over time. The observed data from the experimental huts are overlaid as points to show the capacity of the statistical model to fit the data. (**b**) The relative deterrence of mosquitoes was fit to the initial experimental hut data (overlaid points) and the decay of the deterrence effect was then assumed to mirror that of mortality given that it is infeasible to rotate the sprayed huts and account for local mosquito populations being reduced by effective insecticide (see also Supplementary Fig. S7 and Sherrard-Smith et al.^15^). These trials were completed in northern Ghana where either all walls and ceilings (full IRS) were sprayed (orange), or partial IRS was conducted with pirimiphos-methyl CS spraying the upper walls and ceiling (green), lower walls and ceiling (dark blue), upper walls (red) or lower walls (blue) only. Supplementary Fig. S7 shows how these data are combined, for each spray strategy, to estimate the probable outcome of mosquito feeding attempt including that the mosquito is killed, deterred, blood fed successfully, or repelled without being killed or blood-feeding.

Respective probable outcomes of a mosquito feeding attempt elicited from different spraying scenarios in experimental huts in Ghana suggest that mosquito mortality induced following spraying of lower or upper walls and ceilings is broadly similar to that following full IRS (Fig. 3A). For each of the scenarios, the estimated potency of the deterrence effect is fitted to the observed data which is broadly consistent across IRS strategies, though spraying the upper walls only produced the greatest deterrence (Fig. 3B), but the depreciation in the mortality effect is assumed for each deterrence effect, given the uncertainty in this measurement from experimental hut trial data (discussed elsewhere, see^15^), and remains similar across the full, and each partial IRS strategy (Fig. 3B). Data from a systematic review of experimental huts sprayed with pirimiphos-methyl CS^15^ gives the average probable outcomes of a mosquito feeding attempt under full IRS, and was used to estimate the ratio of probable mosquito blood feeding and exiting consistently across trial arms as this could not be measured with the experimental hut protocol used here (which was adopted to protect volunteers from infectious bites). The IRS strategy-specific probable outcomes of mosquito feeding attempts are shown in the Supplementary File 2 (Supplementary Fig. S7). Taking this approach means that the epidemiological impacts predicted are principally driven by the degree of mortality or deterrence induced by the respective spraying scenarios. The full-IRS (orange lines Fig. 3A) remains most potent at killing mosquitoes but not considerably so and this is reflected in the results (Supplementary Fig. S7).

### Village-scale trial

#### Spray coverage and daily spray output indicators

A total of 3,532 rooms (2,047 in full IRS and 1,485 in partial IRS communities) were found and 3,370 sprayed (1,912 in full IRS and 1,458 in partial IRS communities) in May 2019 (Supplementary Table S4). Spray coverage (percentage of rooms sprayed out of total rooms found) was 93% and 98% in full IRS and partial IRS communities, respectively. The total population protected was 5,040 and 3,875 people in the full IRS and partial IRS communities, respectively.

**Figure 4:**
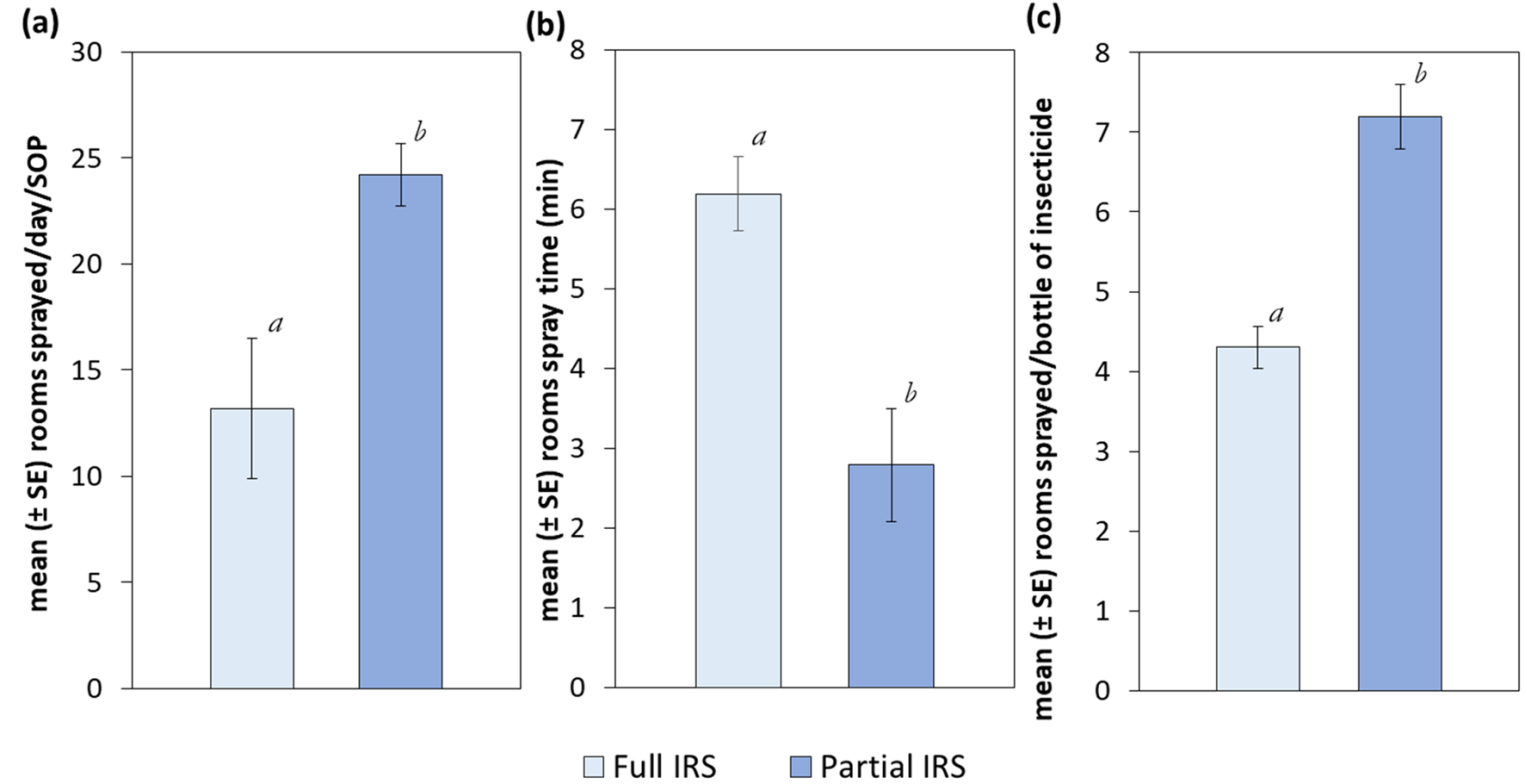
Mean daily spray operator outputs and insecticide consumption in full IRS and partial IRS communities during the 2019 spray campaign in northern Ghana. (**a**) Mean number of eligible rooms sprayed by a spray operator (SOP) in a day, (**b**) Time spent spraying a room, and (**c**) Insecticide consumption expressed as the mean number of rooms sprayed with one bottle of pirimiphos-methyl CS. Different letters on error bars denote statistically significant differences between means at *p* = 0.05.

In the partial IRS communities of Bunbuna, Tum-Tu Zie, and Gupanarigu, SOPs’ average daily output was 26.0, 25.0, and 21.0 rooms, respectively (Supplementary Table S4). However, in the full IRS communities of Banda-ya, Gbullung, and Kpemale, the daily SOP output was 9.0, 11.0, and 19.6 rooms, respectively. Overall, the average daily output in the partial and full IRS communities were 13.2 and 24.2 rooms per day, respectively (Fig. 4A). For reasons that are not clear, coverage was higher in the partial IRS communities (98.2%) than the full IRS communities (93.4%); this could account for some of the differences in the daily SOP output (Supplementary Table S4). The SOPs in the partial IRS communities spent an average 2.8 (2.4–3.1, 95% CI) minutes to spray one room, whereas SOPs in full IRS communities spent 6.2 (5.9–6.6, 95% CI) minutes (Fig. 4B, Supplementary Table S4). One bottle of pirimiphos-methyl CS was used to spray 4.3 rooms in full-IRS communities compared with 7.2 rooms in the partial-IRS communities (Fig. 4C).

### Impact on entomological indices

#### Human biting rates (HBR)

**Figure 5:**
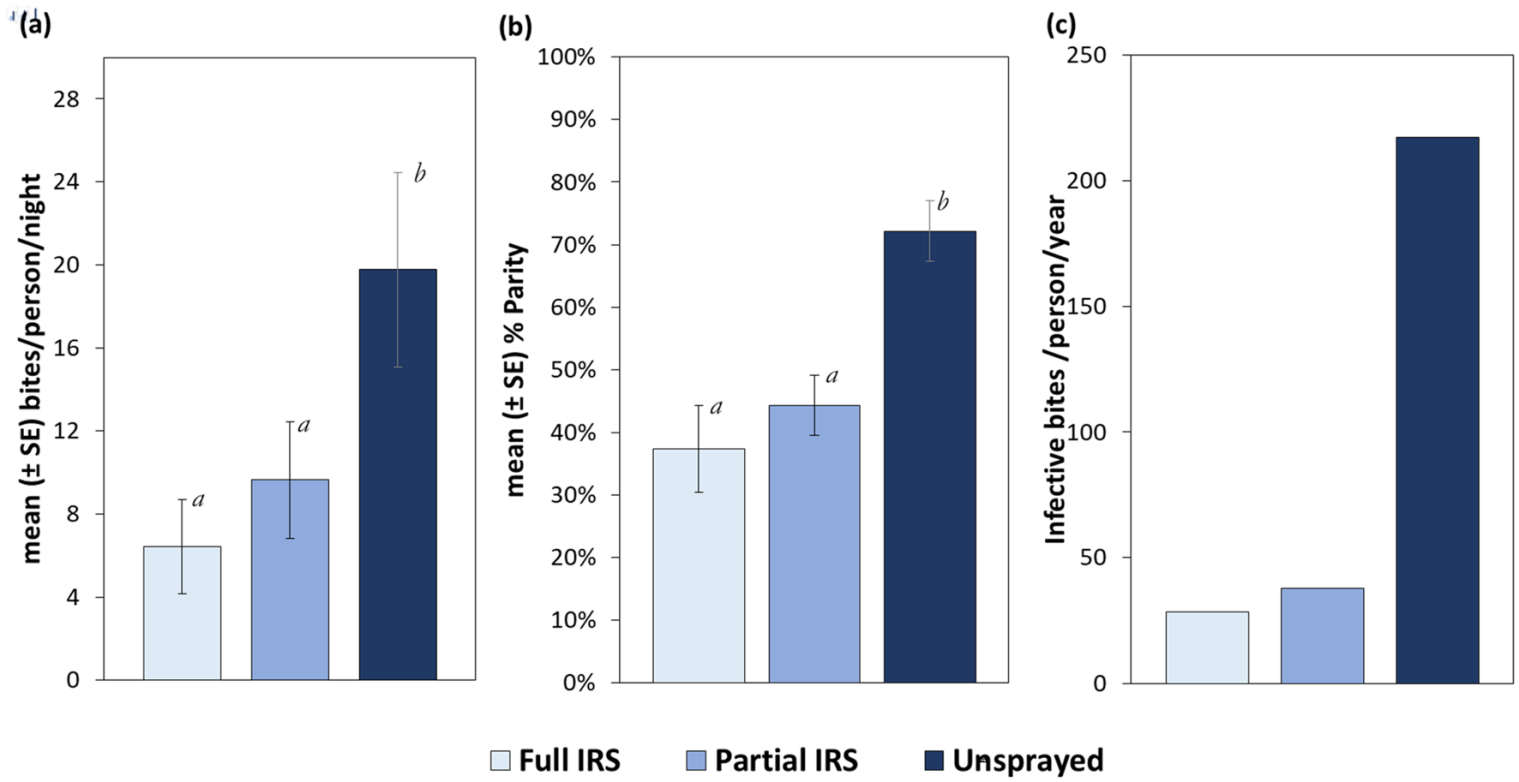
Entomological indices of malaria transmission estimated in full and partial IRS and unsprayed control communities in a village scale trial in northern Ghana, recorded from March–December 2019. (**a**) Human biting rates (HBRs), expressed as mean bites per person per night, of *An. gambiae* s.l. by treatment. (**b**) Mean parity rates of *An. gambiae* s.l. recorded from study sites. (**c**) Entomological inoculation rates (EIR) of *An. gambiae* s.l., expressed as total number of infective bites per person per year, by treatment. The annual EIR was estimated from the sum of monthly EIRs between March and December. Different letters on error bars denote statistically significant differences between means of biting and parity rates at *p* = 0.05. Statistical analysis for EIR were not done.

A year-to-year comparison of the post-spray HBRs (June to December) of *An. gambiae* s.l. shows there was a 19% decrease in the HBR of *An. gambiae* s.l. from communities that received full IRS compared to a 5% decrease in partial IRS communities in 2019 as compared to HBRs in 2018, when all the communities were fully sprayed (Supplementary Fig. S4). The difference in mean HBRs in the full IRS communities between years was significant (*p* < 0.001), but the difference in HBRs in partial IRS communities was not (*p* = 0.791). Overall, the mean HBR of *An. gambiae* s.l. in both fully sprayed (6.4 bites/person/night (b/p/n)) and partially sprayed (9.6 b/p/n) sites was significantly lower (*p* < 0.001) than in the unsprayed control sites (19.8 b/p/n) in 2019. Controlling for fixed effects for month, random effects for community, household, and place of collection (indoor/outdoor) of data collection, the differences between partially and fully sprayed areas were not significant (*p* = 0.339) (Fig. 5A).

#### Parity rates

The mean parity rates in the full IRS communities declined from 0.43 in 2018 to 0.37 in 2019 (*p* = 0.99), while the rates in partial IRS communities increased from 0.32 in 2018 (when fully sprayed) to 0.44 in 2019 (Supplementary Fig. S5). However, this increase was not significant (*p* = 0.60). Similarly, the parity rates in the unsprayed control sites increased from 0.68 in 2018 to 0.72 in 2019 (*p* = 0.94). The post-spray mean parity of *An. gambiae* s.l. in 2019 in both fully (39%) and partially sprayed communities (45%) were significantly lower (*p* < 0.001) than the control sites (66%), but the difference between parity rates in partially and fully sprayed areas was not significant (*p* = 0.66) (Fig. 5B).

#### Entomological inoculation rate

The annual EIR, an estimated risk of malaria transmission, was higher for the control sites at 217.4 ib/p/yr, compared with the full IRS and partial IRS sites, with estimates of 28.4 ib/p/yr and 37.7 ib/p/yr, respectively (Fig. 5C, Supplementary Table S5).

#### Pirimiphos-methyl CS spray quality, residual efficacy and fumigant effect

Spray quality, as measured by mosquito (*An. gambiae* Kisumu strain) mortality at 24 h following exposure to sprayed surfaces 1–3 days after spray, ranged between 98 and 100% on all wall surface types (mud, cement, and wood) across all sites (Supplementary Fig. S6), indicating no under-dosing of insecticide. Subsequent bioassays conducted monthly showed that pirimiphos-methyl CS remained effective above the cut-off mosquito mortality level (80% 24-h post-exposure mortality) for 6–9 months post-IRS, depending on the type of surface sprayed and the site (Supplementary Fig. S6). Despite the variations in the mortality on the different surfaces, the differences were not significant (*p* = 0.622). A fumigant effect of pirimiphos-methyl CS was also observed at all sites, with some variations, but the differences were not significant (*p* = 0.97). However, this effect generally decreased after two months post-spray across the sites tested. There were no significant differences between the decay rates of sprayed insecticide in partial and full-IRS communities (p = 0.21).

#### Predicting epidemiological impact of partial IRS with pirimiphos-methyl CS

**Figure 6:**
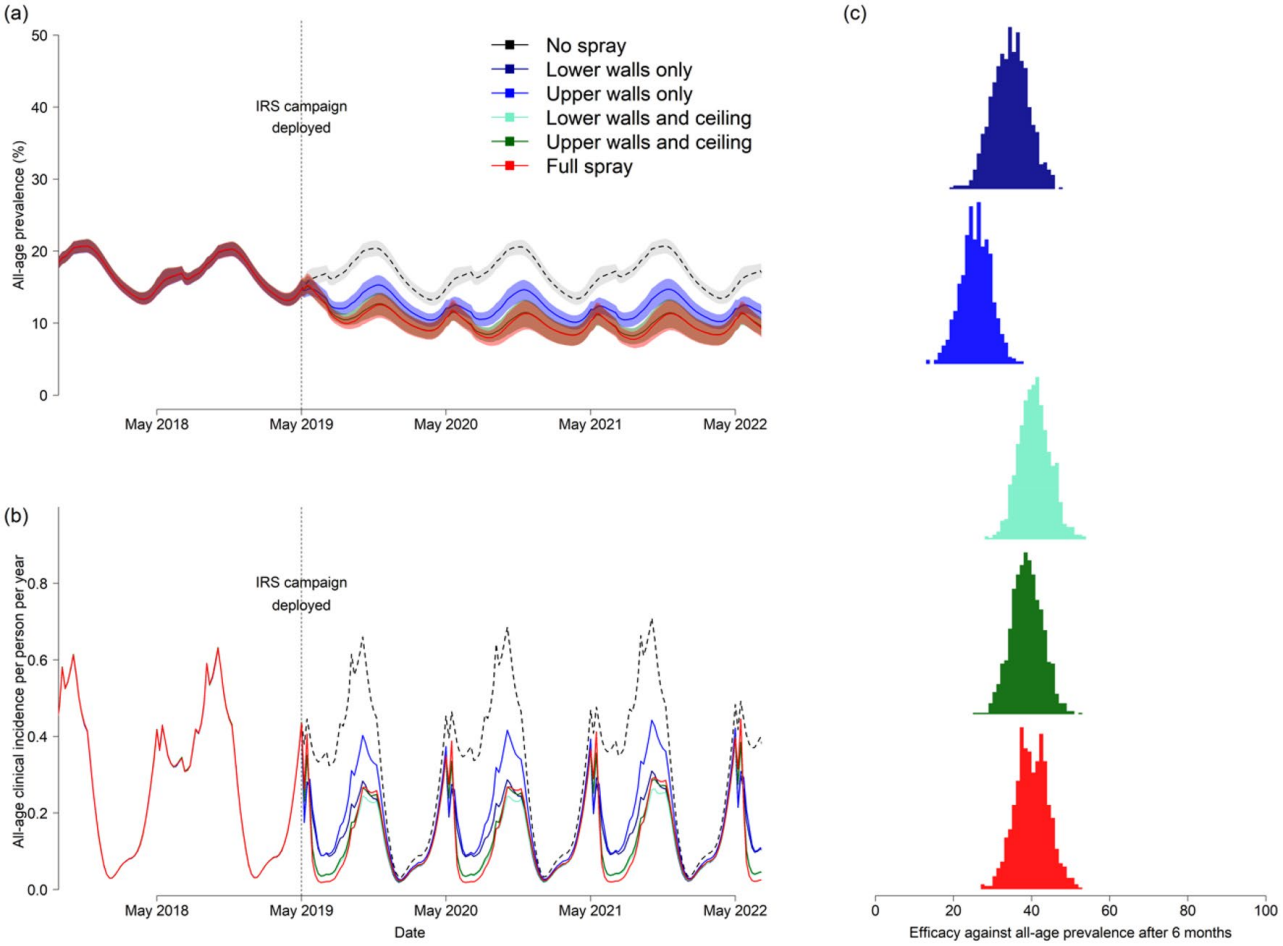
Model predicted epidemiological impact of partial versus full IRS with pirimiphos-methyl CS. In all panels, the different partial IRS scenarios are shown as: lower walls only (dark blue), upper walls only (light blue), lower walls and ceiling (light green), upper walls and ceiling (dark green), or full spray (red). The model uncertainty (95% uncertainty intervals) is shown in panel (**a**) and (**c**) around the mean (line, panel (**a**)). Panel (**a**) shows the model simulated all-age malaria prevalence (mean, lines, and 95% uncertainty intervals (shaded polygons)) over time with the spray campaign taking place annually from May 2019 onward. The no spray counterfactual (grey dashed line and shaded polygon) is used to compare with all spray strategies to estimate the efficacy against prevalence (as shown in Table 1 and panel (**c**) at 6 months after the first spray campaign). (**b**) The corresponding model estimates of all-age clinical incidence of malaria (mean estimate over time for clinical cases per person per year) for the same trial arms and counterfactual simulation. The vertical dotted lines on panel (**a**) and (**b**) in May 2019 indicate the time when spray was deployed. (**c**) The model predicted efficacy against all-age malaria prevalence relative to no spray campaign (grey dashed lines on panels (**a**)) at six months post-IRS for each spray strategy respectively. The uncertainty is carried through from the model simulations and parameter uncertainty in IRS efficacy estimates (Supplementary File 2) and shown by the width of the histogram for each spray strategy.

The probability outcomes of a mosquito feeding attempt, as estimated from the experimental hut data analysis and systematic review (Fig. 3), were used to adjust the transmission model parameterization, and infer epidemiological impact (Fig. 6). Figure 6A demonstrates this impact first for all-age prevalence over time, simulating annual rounds of full or partial IRS, and this is reflected in the clinical incidence (Fig. 6B). The percentage reduction in malaria prevalence and incidence achieved by each IRS strategy was estimated relative to no spray campaign. Contrasting impacts between IRS strategies are driven by the mortality and deterrence effect given the way that the experimental hut data were used to parameterize the model. The model predicts that the relative efficacy against all-age prevalence after six months (Fig. 6C) is 40.0% (32.2–48.4%, 95% uncertainty intervals, UI) when a full IRS campaign is completed, but the equivalent efficacies for a partial IRS campaign where only the lower walls and ceiling (40.8% (33.2–49.2%, 95% UI)) or upper walls and ceiling (38.8% (31.1–47.1%, 95% UI)) are sprayed are predicted to be as effective. Spray campaigns targeting only the walls were less effective. Table 1 provides the full results for each spray strategy.

**Table 1:** Modelled estimates of the epidemiological effectiveness of partial versus full IRS with pirimiphos-methyl CS relative to no spraying in a setting similar to northern Ghana.

Spray scenario	Clinical cases averted per 1000 people in 1st year since spraying		% reduction in all-age prevalence relative to no spraying (in months, post-spray)			
	All-age	Under 5 years	2 months	4 months	6 months	8 months
No spray	(counterfactual)		(counterfactual)			
Lower walls	161 (131–194)	226 (158–293)	28.2 (21.4–34.2)	35.8 (28.3–43.1)	34.6 (26.1–43.5)	33.4 (24.2–43.3)
Upper walls	129 (103–154)	182 (124–241)	27.2 (20.6–33.2)	29.3 (21.9–36.5)	25.7 (17.9–33.3)	23.4 (15.6–30.6)
Lower walls and ceiling	182 (157–207)	254 (199–313)	34.8 (29.2–40.1)	43.6 (37.3–49.7)	40.8 (33.4–48.9)	38.1 (30.1–47.1)
Upper walls and ceiling	176 (150–203)	246 (186–305)	34.3 (28.6–39.7)	42.0 (35.4–48.2)	38.7 (31.0–46.6)	35.9 (27.6–44.5)
Full spray	180 (155- 205)	250 (192–305)	36.6 (30.7–41.9)	44.4 (37.5–51.1)	40.0 (31.7–48.4)	36.3 (28.0–45.5)

### Cost savings and feasibility

#### Overall savings from the spray operations and insecticide costs

A daily target of 18 rooms per SOP has been used historically to plan for a 30-day spray campaign in northern Ghana. The data suggests a mean SOP daily output (rooms/SOP) of 24.2 rooms per day for partial IRS (Fig. 4A), which translates into approximately 22.3 days to complete a partial IRS campaign. The reduced number of days spent spraying would therefore result in savings on the daily wages for teams directly involved in spraying (SOPs, team leaders, washers, and water fetchers), as well as for meals and transportation (vehicles rented and fuel costs). The direct daily expenses related to spraying days for all 7 districts covered with full IRS in northern Ghana in 2018 was approximately $311,576.80 for a 30-day campaign. It is estimated that a 22.3-day partial IRS campaign in these districts would cost $231,605.42, representing approximately 25.7% savings ($79,971.38) on operational costs (Table 2; Supplementary Table S6).

**Table 2:** Estimated savings on direct IRS operational expenses and insecticide costs based on a 30-day full IRS campaign and a 22.3-day partial IRS campaign based on the 2018 PMI VectorLink Ghana IRS campaign.

Cost indicator	Cost of full IRS campaign *†*	Estimated cost of partial IRS Campaign	Projected savings	% Savings
**Operational cost**				
Daily wages of spray teams	$133,206.96	$99,017.17	$34,189.79	
Meals	$38,304.19	$28,472.78	$9,831.41	
Transport & related cost	$140,065.65	$104,115.47	$35,950.18	
		Sub-total	$79,971.38	25.7%
Insecticide cost*				
Cost of IRS Insecticide	$1,061,157.36	$644,701.99	$416,455.37	39.2%
		**Overall savings**	**$496,426.75**	**36.2%**

*Cost of Actellic CS, 30% a.i., Syngenta used for this estimation is $16.19 price based on 2018 cost (shipping and insurance inclusive).

*†Estimated cost is based on 298,701 rooms sprayed and 65,544 Actellic CS, 30% a.i. bottles used during the 2018 IRS (full IRS) campaign.*

In 2018, a total of 298,701 rooms were sprayed across all 7 districts in northern Ghana, using 65,544 bottles of pirimiphos-methyl CS. The average number of rooms sprayed per bottle was 4.6. Based on the mean insecticide consumption rate of 7.2 rooms per bottle in the partial IRS communities (Fig. 4C), it was estimated that if these districts had received partial IRS in 2018, approximately 25,723 bottles of insecticides would have been saved. This translates to a cost savings of $416,455.37 (39%) on insecticides alone compared with full IRS (based on a price of $16.19 per bottle of Actellic CS, 30% ai, Syngenta in 2018) (Table 2; Supplementary Table S7). Overall a total of $496,426.75 (about 36% of the direct cost of field operations) could have been saved from reduced costs of insecticide and operations if all districts in northern Ghana were sprayed partially in 2018.

#### Cost effectiveness of partial spraying pirimiphos-methyl CS IRS

The cost per person of full and partial IRS campaigns as estimated directly from the village-scale trial were $5.74 and $4.94, respectively. Using the transmission model, the estimated cost per clinical case averted per year for a full IRS campaign in a region representative of northern Ghana would be $1.04 ($0.93–$1.14, 95% uncertainty intervals, UI) compared to $0.87 ($0.78–$0.97, 95% UI) for a partial IRS campaign.

#### Community perceptions of partial IRS

Of the 63 respondents interviewed seven months after spraying in the partial IRS communities, 52 expressed their views about the perceived quality of partial IRS (Table 3). In comparison with the full IRS, they received in the previous year, 48% (26/54) felt that it was as effective as the full IRS, whereas 50% (27/54) thought it was not as effective as the previous years’ full IRS. Only 2% (1/54) of respondents felt it was better than full IRS because there was no run-off from the wall as with the full IRS. Of the 63 respondents, 60% (38/63) reported that they would agree to have their homes partially sprayed in the next spray round. Only 16 respondents provided reasons for this decision; 12 based their decision on the opinion that it was the same as full IRS, while the remaining four reported that it was because they thought it killed other insects. Approximately 40% (38/63) of the respondents expressed concerns with partial IRS and were not ready to accept partial IRS. Their concerns included the perception that they observed more mosquitoes in 2019 than after receiving full IRS in 2018, (74%; 22/31 respondents). Others (23%, 7/31 respondents) complained that they did not see other insects such as cockroaches and crickets dying. However, none of the respondents in the partial IRS communities perceived that it would not be effective against mosquitoes or malaria. A few (6%, 2/31) of the respondents wanted all other places (including toilets, stores, and animal shelters) to be sprayed in addition to their sleeping rooms (Table 3).

**﻿Table 3:** Perceptions of community members on partial IRS, as determined through interviews conducted seven months post-spray.

Variable/Indicator	Full IRS		Partial IRS	
	N	%	N	%
**Total Number of Respondent Interviewed**	**125**		**63**	
Thoughts on the partial IRS done this year*		n/a	54	
Effective/Prevent visits to the hospital/malaria				48%
Not effective/more mosquitoes/does not kill other insects				50%
Better than full spray/less run-off from wall				2%
**Respondents that WOULD accept partial IRS**		46%		60%
**Responses to why respondents WOULD accept partial spraying**	48		16	
Would accept if partial IRS is effective		21%		n/a
Would allow project to expand		21%		
Will take less time		18%		
Reduce smell		12%		
Reduce insecticide run-off & cleaning		15%		
Reduced items to pack out		3%		
Less insecticide danger/No itching		9%		
Same as full spray*		n/a		75%
Killed other insects*				25%
**Responses to why respondents WOULD NOT accept partial spraying**	37		31	
Full IRS more effective/lasts longer/Prefer full IRS*		16%		10%
Mosquitoes will rest on unsprayed surfaces /Felt there were more mosquitoes*		19%		74%
Will not kill other insects/did not see other insects die*		32%		23%
Will not be effective against mosquitoes/malaria		32%		0%
All other places (Animal shelter/stores) must be sprayed in the partial spray communities*		0%		6%

*Questions and responses for partial IRS communities only.

In the full IRS communities, 46% (58/125) reported that they would agree for their homes to be partially sprayed in the next spray round. Of these, 48 provided reasons for their decision. Some (21%) indicated that the decision would be dependent on the efficacy demonstrated from the village trial and a similar proportion (21%) indicated that they would accept partial IRS because it was going to allow for expansion of the program in other areas. About 12% (6/48) were willing to accept partial IRS because they thought it would reduce the smell of the insecticide and the remaining 18% (9/48) of respondents thought that SOPs would spend less time spraying their home.

The majority (54%, 67/125) of respondents from the full IRS communities expressed that they would not accept partial IRS. Of those (30%, 37/125) that gave reasons for this decision not to accept partial IRS 32% (12/37) thought that IRS should kill other insects and assumed that partial IRS will not kill other insects. A similar proportion were concerned about partial IRS not offering them the needed protection from mosquito bites and malaria. About 16% (6/37) preferred full IRS because they thought it was more effective and would last longer, while 19% (7/37) felt that the mosquitoes that enter to feed could still rest on the unsprayed surfaces in the room (Table 3).

## Discussion

While IRS is a proven vector control intervention for malaria control, the costs of insecticides and field operations pose challenges to its scalability and sustainability. Innovative approaches to the application of IRS are needed to reduce costs and/or streamline operations while simultaneously providing a similar level of protection in terms of mosquito mortality. In this study, we have demonstrated proof-of-principle for partial IRS in experimental huts and identified a promising partial IRS approach (spraying only upper walls and ceilings) that was further assessed in a village-scale trial in northern Ghana where it was more cost-effective yet equally effective as full IRS. Using these data to parameterize a malaria transmission model, we further showed that the potential epidemiological impact of partial IRS could be comparable to that of full IRS in settings similar to northern Ghana.

Prior to spraying with pirimiphos-methyl CS, most mosquitoes were captured resting inside of experimental huts on ceilings, though a small proportion were captured from the cement walls, suggesting that ceilings are the preferred resting location of *An. gambiae* s.l. (mostly *An. gambiae* s.s.) in this area (Supplementary Fig. S3). This is in line with previous observations from northern Ghana, where *An. gambiae* s.s. was found to rest more frequently on ceilings and upper parts of the walls of grass thatched houses^19^, and in southern Ghana where 75.9% of *An. gambiae* s.s., 73.6% *An. funestus*, and 58.2% *An. coluzzii* were found resting above 200 cm from the floor of houses, on either the walls or ceiling^37^. Similar resting preferences have been reported for *An. gambiae* s.l. and *An. funestus* in Burkina Faso^38^ and *An. funestus* s.s. in Zimbabwe^39^, while results from Tanzania were mixed^40^. In Kenya, *An. gambiae* s.l. appeared to prefer to rest on the lower section of walls^41^. Malaria vector resting behavior can vary substantially by species and geographical location, as well as house/hut design^42^ and temperature, depending on the height of wall or room^43^. Thus, determining local vector species behavior will be critical in assessing whether implementation of partial IRS as done in this study would be effective for malaria vector control.

Partial IRS of experimental huts, specifically when either the upper or lower walls and ceiling were sprayed, demonstrated a similar level of efficacy as fully sprayed experimental huts (about 10% difference in mortality). This suggested that although *An. gambiae* s.l. prefers to rest on the ceiling, mosquitoes were not avoiding sprayed surfaces in the partially sprayed experimental huts. It should be noted that while pirimiphos-methyl CS is known to have both contact and fumigant effects^44^, the fumigant effect is short-lived, lasting roughly two months after spraying before declining significantly^45^. Therefore, while it is possible that even without direct contact to sprayed surfaces, mosquitoes could still receive a lethal airborne dose of the sprayed insecticide, it is unlikely that the fumigant effect of pirimiphos-methyl CS played a major role in the efficacy observed in this study.

The probable outcome of a mosquito feeding attempt indoors is assumed to end with either the mosquito successfully feeding, being repelled (deterred from entering from the outside or exiting the house without feeding) or being killed. The presence of insecticide applied through IRS will alter this probability relative to a scenario with or without ITNs^46^ and these effects can be incorporated into malaria transmission models to understand how entomological effects translate to public health impact^16,17^. Based on the experimental hut data generated here, it is expected that the probable outcomes (being killed or repelled) of a mosquito feeding attempt elicited from partially (lower or upper walls and ceilings) or fully spraying huts in Ghana would be similar. However, due to the low sample size of blood fed mosquitoes across all spraying scenarios (a consequence of the unholed nets used to protect volunteer sleepers in the trials), no predictions could be made regarding differences in blood feeding success elicited from partially or fully sprayed huts.

Given these results, and potential cost savings from reduced insecticide quantities and operational costs, particularly the perceived benefit of not having to remove as much furniture from houses, spraying of upper walls and ceilings was selected as the best approach for further assessment of partial IRS in comparison with full IRS and no IRS in a village-scale trial in eight communities in northern Ghana. No significant differences in *An. gambiae* s.l. HBRs and parity rates were observed between partial and full IRS communities, though all entomological outcomes were significantly lower in both IRS scenarios than in the unsprayed communities. Further, the relatively lower annual EIRs observed in both full and partial IRS communities in comparison with the unsprayed sites (where the sum of monthly EIRs was 217 ib/p/yr) suggests a similar level of suppressing the risk for malaria transmission among the two spraying scenarios. Further, the model predicts that the benefit in terms of reduction in all age malaria prevalence achieved by the full spraying is broadly matched where the ceiling and a section of the walls are sprayed, hence indicating a potentially equivalent epidemiological effect of partial IRS.

A small number of previous studies have demonstrated comparable entomological and epidemiological effectiveness of other partial IRS approaches for malaria control, but there is little information available on the cost-effectiveness of these approaches. “Band spraying” has been shown to be effective in multiple settings: with DDT against *An. sacharovi* and *An. superpictus* in Lebanon^14^ and *An. minimus* in Taiwan^47^, in addition to reducing parasite rates, as well as with bendiocarb against *An. albimanus* in Mexico^12^ and *An. flavirostris* in the Philippines^13^. A similar partial IRS approach where the lower half of walls are targeted has been shown to be effective against *Aedes aegypti*, the main vector of dengue, in Mexico^48^. These findings were instrumental in the development of new guidelines from the Pan American Health Organization (WHO) for IRS in urban areas which targets the lower walls based on the resting behavior *Ae. aegypti*^49^. While the band spraying approach showed costs savings similar to what was estimated for partial IRS of pirimiphos-methyl CS in this study, approximately 30% of total expenses in Lebanon and 25.5% of operational costs in Taiwan, respectively, there are no indications that the programs adopted this alternative approach for IRS, though both countries were declared malaria free in the 1960s. Additionally, selective spraying with fenitrothion in Indonesia also reduced malaria and the vector population, primarily *An. aconitus,* to levels as low as full spraying, and reduced the cost by 68%^50^.

In addition to the demonstrated entomological effectiveness and predicted epidemiological impact, partial IRS as implemented in this setting is expected to have considerable cost savings, roughly 33%, or nearly $500,000, as compared to full IRS. This is primarily attributed to a 39% reduction in insecticide costs and 26% decrease in operational expenses. These cost savings would enable spraying of an additional 36,000 rooms (12% increase) in northern Ghana. Moreover, the estimated cost per clinical case averted per year for a partial IRS campaign is estimated to be $0.17, or roughly 12%, less than for a full IRS campaign. However, it is apparent from the qualitative surveys that in order to scale-up, special messaging is needed to explain the benefits in order to improve acceptance among homeowners.

This study has empirically demonstrated that the amount of insecticide sprayed, and operational costs can be reduced with a partial IRS approach without compromising the entomological efficacy of IRS. The epidemiological impact of partial IRS, as predicted by mechanistic modeling, is also expected to be equivalent to that of full IRS. However, there is need for proper community engagement to promote, and potentially modify implementation of a partial IRS approach if it is to be implemented at scale. Further large-scale investigations, including empirically measuring impact on epidemiological outcomes in randomized control trials would further build the body of evidence for scale-up and adoption of this cost-effective approach for IRS by national malaria control programs.

## Supplementary Information

Supplementary Information.

## Data Availability

The datasets used in this study are available from the corresponding author on reasonable request.
