# Supplementary material for: Partial indoor residual spraying with pirimiphos-methyl as an effective and cost-saving measure for the control of Anopheles gambiae s.l. in northern Ghana
Source: Sci Rep. 2021 Sep 10;11:18055. doi: 10.1038/s41598-021-97138-1 (PMC8433436; doi:10.1038/s41598-021-97138-1)
Supplement: Supplementary file 1 — Supplementary Information. [file 41598_2021_97138_MOESM1_ESM.docx]

SUPPLEMENTARY INFORMATION

**Partial indoor residual spraying with pirimiphos-methyl as an effective and cost-saving measure for the control of *Anopheles gambiae* s.l. in northern Ghana**

**Sylvester Coleman^1^**^,*^, Yemane Yihdego^2,*^, Ellie Sherrard-Smith^3^, Churcher S. Thomas^3^, Dereje Dengela^2^, Richard M Oxborough^2^ , Samuel K. Dadzie^4^, Daniel Boakye^4^, Frank Gyamfi^1^ , Kwasi Obiri-Danso^5^, Ben Johns^2^, Lilly V Siems^2^, Bradford Lucas^2^, Jon Eric Tongren^6^, Sixte Zigirumugabe^6^, Dominic Dery^6^, Christen Fornadel^7^, Kristen George^7^, Allison Belemvire^7^, Jenny Carlson^7^, Seth R. Irish^8^, Jennifer S. Armistead^7^, Aklilu Seyoum^2,*^

Supplementary File 1

**Supplementary Figures and Tables from the experimental hut and village scale trials on partial indoor residual spraying in northern Ghana**


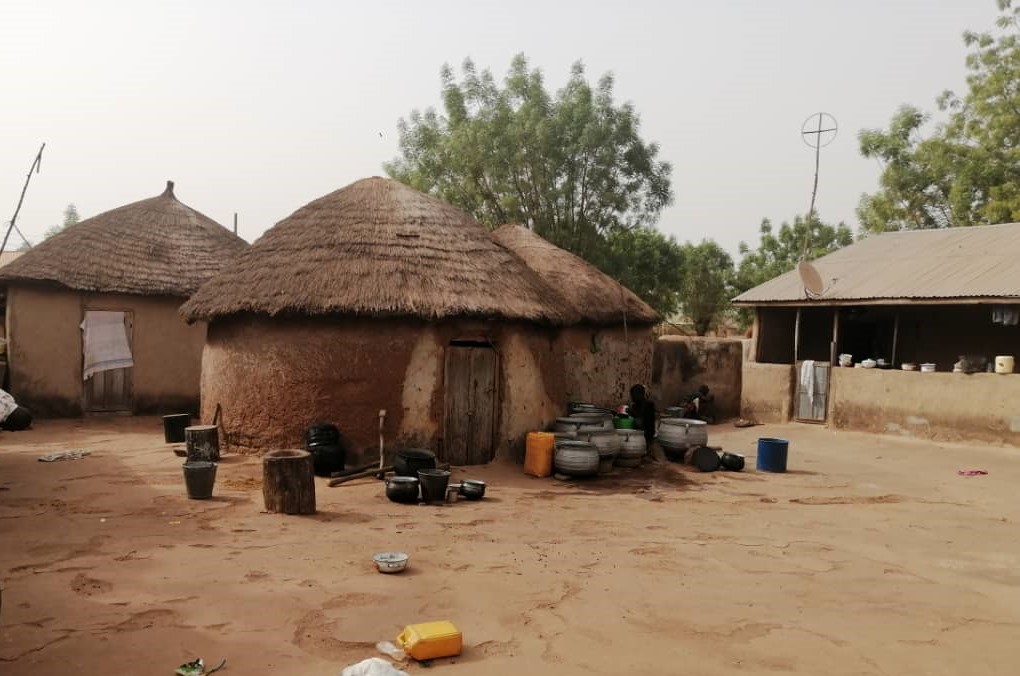


Supplementary Figure S1: A typical house structure in rural northern Ghana.


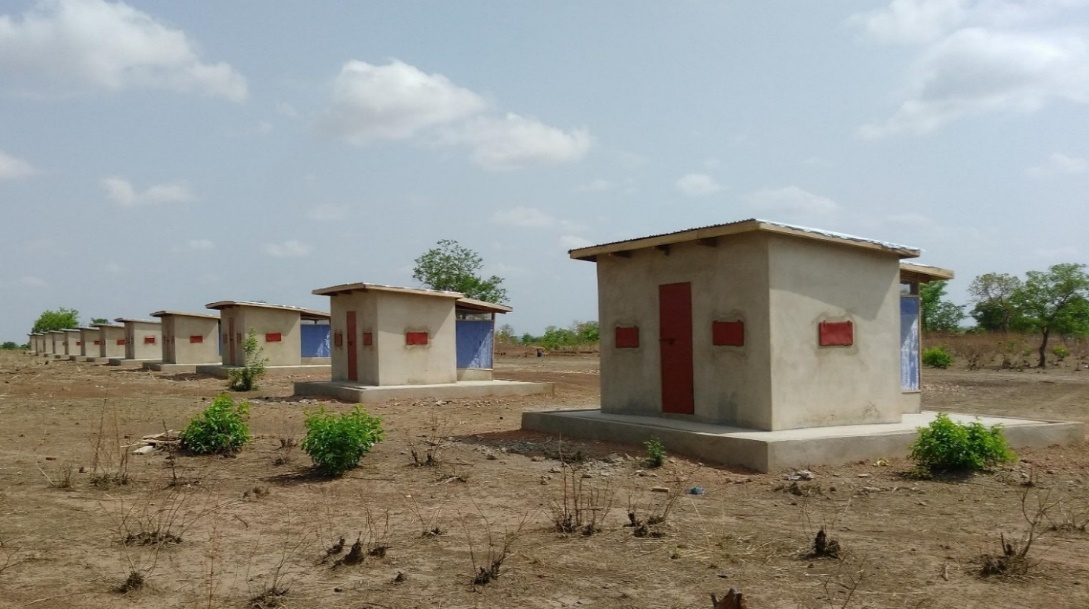


Supplementary Figure S2. Experimental huts for evaluation of partial IRS at Kulaa in Tamale Metropolitan District, northern Ghana.

Supplementary Figure S3: Percentages *of Anopheles gambiae* s.l. collected by resting location in experimental huts prior to spray from mouth aspirator collections. Percentages were calculated from all mosquitoes collected inside huts and the veranda.


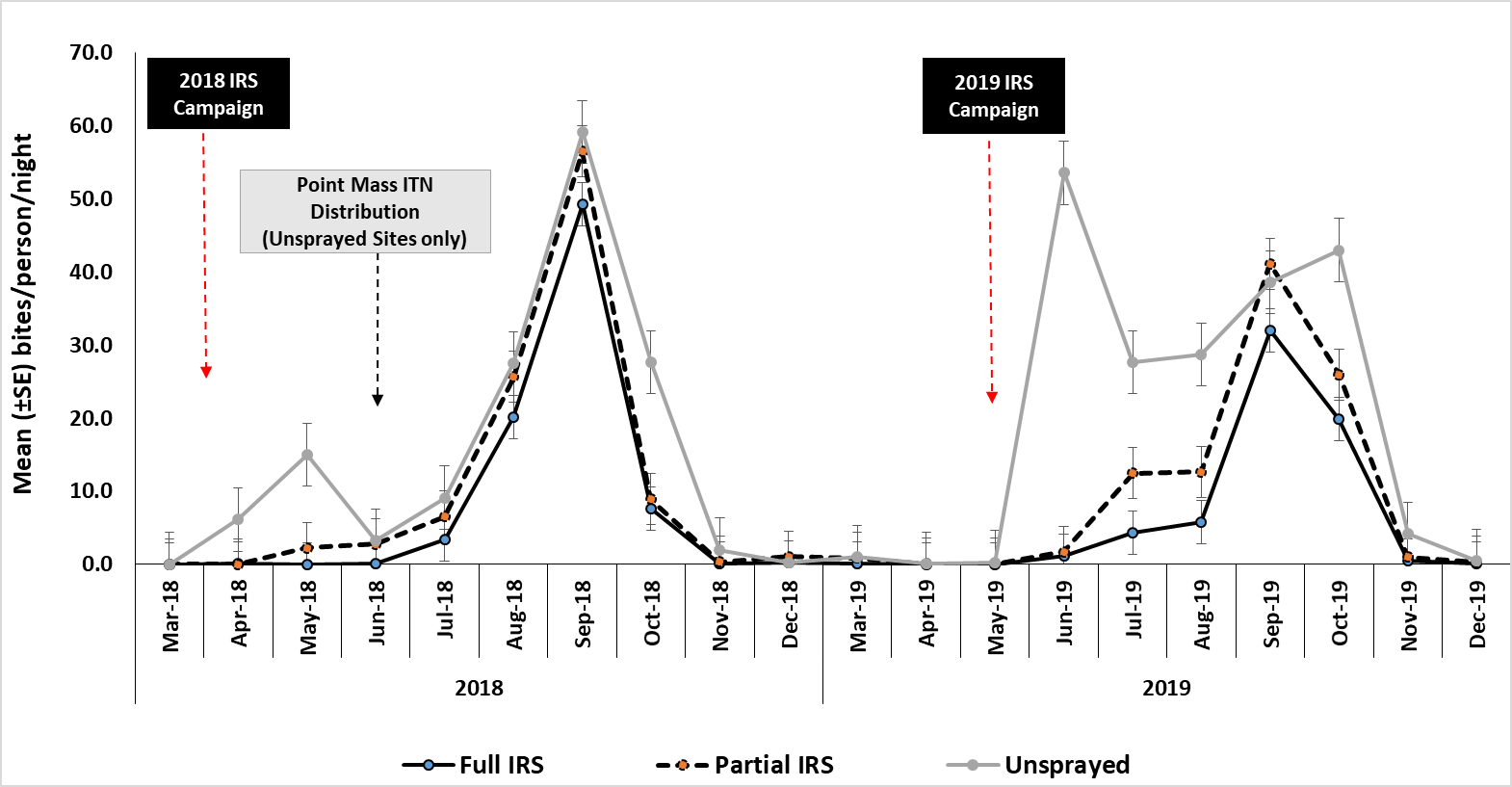

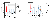


Supplementary Figure S4: Mean Daily Human Biting Rates (HBRs) of *An. gambiae* s.l. in northern Ghana, by month, March 2018 – December 2019. A year-to-year comparison shows that the HBRs of *An. gambiae* s.l. from both the full IRS (solid black line with blue dot) and partial IRS (dashed black lines with red dot) communities were lower in 2019 as compared to 2018 when all the communities were fully sprayed. In 2019, the HBRs of *An. gambiae* s.l., from both full and partial IRS communities were lower than that from unsprayed communities (solid gray line).


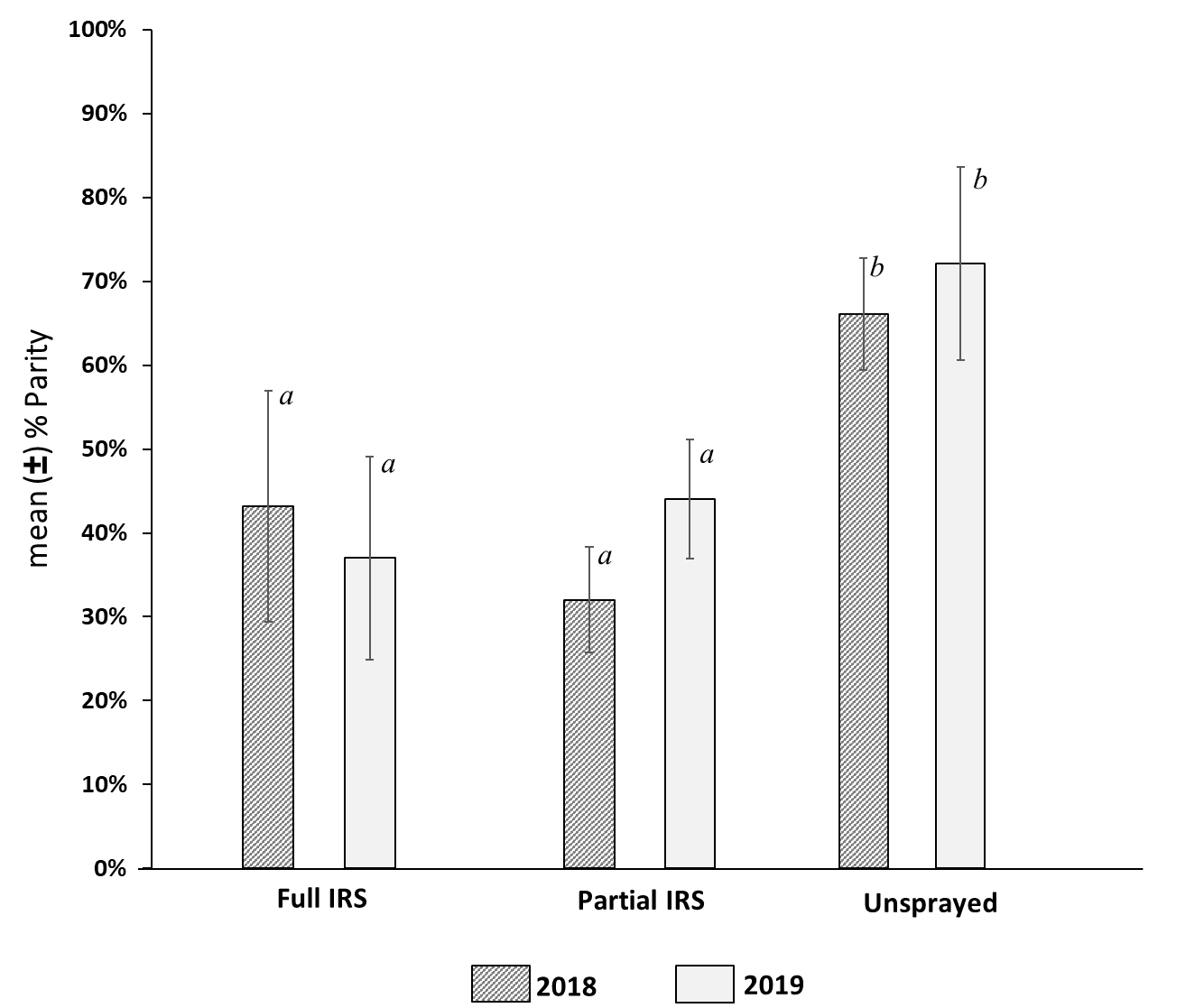


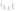


**Supplementary Figure S5: Mean parity rates of *An. gambiae* s.l. in full IRS, partial IRS, and unsprayed control communities, March 2018–December 2019.** A year-to-year comparison of the mean parity of *An. gambiae* s.l. shows a decline in parity rates in the full IRS communities in 2019, while the rates in partial IRS communities increased in 2019. However differences between parity rates in partially and fully sprayed areas was not significant. Parity rates in unsprayed sites remained significantly higher than both partial and full IRS sites in both 2018 and 2019.

**Supplementary Figure S6: Spray quality, residual efficacy, and fumigant effect of pirimiphos-methyl CS in full and partial IRS communities.** Spray quality, as measured by mosquito (*An. gambiae* Kisumu strain) mortality at 24 hours following exposure to sprayed surfaces in cone bioassays at time zero (1-3 days post-spray) showed 98-100% mortality across surfaces at all sites. Subsequent monthly bioassays showed that pirimiphos-methyl CS remained effective above the 80% 24-hour post-exposure mortality threshold (red dashed line) for 6–9 months post-IRS, depending on the type of surface sprayed and site. A fumigant effect of the insecticide was observed at all sites but rapidly declined after one (T1) to two months (T2). Red-dashed line represents the 80% WHO threshold mortality.

Supplementary Table S1: History of IRS and insecticides used in the village-scale trial communities in northern Ghana, 2008 – 2019.

| **Districts** | **Sentinel Sites** | **Insecticide Spray History** | | | | | | | | | | |  |
| --- | --- | --- | --- | --- | --- | --- | --- | --- | --- | --- | --- | --- | --- |
|  |  | **2008** | **2009** | **2010** | **2011** | **2012** | **2013** | **2014** | **2015** | **2016** | **2017** | **2018** | **2019** |
| BYD | Bunbuna & Kpemale | NSp | NSp | NSp | ACy | ACy | PM | PM | PM | PM | PM | PM | PM |
| GUD | Banda-ya & Tum-Tu Zie | ACy | ACy | DM | ACy | ACy | NSp | NSp | NSp | NSp | PM | PM | PM |
| KUD | Gbullung & Gupanarigu | ACy | ACy | DM | ACy | ACy | NSp | NSp | PM | PM | PM | PM | PM |
| TML | Kulaa & Tugu (comparison sites with no history of IRS) | Control | Control | Control | Control | Control | Control | Control | Control | Control | Control | Control | Control |
| *Note:* NSp=not sprayed; DM=deltamethrin; ACy=alpha-cypermethrin; PM=pirimiphos-methyl | | | | | | | | | | | | |  |

Supplementary Table S2: Status of *An. gambiae* s.l. for the nine rounds of post-spray collections from experimental huts, by treatment types at time of collection and after a 24-hour holding period

| **Hut Treatment** | **Total collected** | **#Alive (morning)** | **# Dead (morning)** | **# Alive  (at 24 hr)** | | **# Dead  (at 24 hr)** | **% Total Mortality*** | **% Blood fed**** |
| --- | --- | --- | --- | --- | --- | --- | --- | --- |
| Unsprayed | 1546 | 1530 | 16 | | 1322 | 208 | 14.5 | 33.5 |
| Fully sprayed | 1042 | 429 | 613 | | 242 | 187 | 76.8 | 25.5 |
| Lower wall only | 997 | 765 | 232 | | 541 | 224 | 45.7 | 26.9 |
| Lower wall + Ceiling | 870 | 475 | 395 | | 277 | 198 | 68.2 | 25.9 |
| Upper wall only | 815 | 646 | 169 | | 482 | 164 | 40.9 | 27.2 |
| Upper wall + Ceiling | 1047 | 619 | 428 | | 351 | 268 | 66.5 | 31.5 |

*% Total mortality refers to total number of dead mosquitoes at the time of collection in the morning and after 24 hrs holding period; ** % blood fed is the proportion of blood fed mosquitoes out of the total collected from each hut

Supplementary Table S3: Comparison of *An. gambiae* s.l. mortality post-intervention in sprayed and unsprayed experimental huts using adjusted negative binomial regression with random effects for volunteer sleeper, hut, and time since spray

| Hut Treatment | Adjusted negative binomial model | | | |
| --- | --- | --- | --- | --- |
|  | IRR | P>\|z\| | LCI | UCI |
|  |  |  |  |  |
| Fully sprayed | Comparator | |  |  |
| Unsprayed | 0.19 | <0.001*** | 0.16 | 0.23 |
| Lower wall + Ceiling | 0.90 | 0.15 | 0.77 | 1.04 |
| Upper wall only | 0.57 | <0.001*** | 0.48 | 0.68 |
| Upper wall + Ceiling | 0.89 | 0.13 | 0.77 | 1.03 |
| n (number of hut - nights) | 928 |  |  |  |
| Log likelihood | -1,426 |  |  |  |

IRR: Incidence rate ratio; LCI: Lower bound of the 95% confidence interval; UCI: Upper bound of the 95% confidence interval.

Supplementary Table S4: Summary of IRS operational outcomes in the full and partial IRS communities in northern Ghana.

| District | Bunkpurugu  -Nakpanduri | | |  | Gushegu | |  | Kumbungu | | | Mean of Indicators | |  |
| --- | --- | --- | --- | --- | --- | --- | --- | --- | --- | --- | --- | --- | --- |
| Study Site  (Spray Treatment) | Kpemale  (Full-IRS) | | Bunbuna  (Partial-IRS) |  | Banda-ya  (Full-  IRS) | Tum-Tu Zie  (Partial-IRS) |  | Gbullung  (Full-IRS) | | Gupanarigu  (Partial-IRS) | Full  -IRS | Partial-IRS |  |
| *Spray Coverage* | | | | | | | | | | | | |  |
| Rooms found | 605 | | 791 |  | 340 | 151 |  | 1,102 | 543 | | 2,047 | 1,485 | |
| Rooms sprayed | 588 | | 789 |  | 305 | 150 |  | 1,019 | 519 | | 1,912 | 1,458 | |
| Spray coverage (%) | 97.2% | | 99.7% |  | 89.7% | 99.3% |  | 92.5% | 95.6% | | 93.4% | 98.2% | |
| Population protected | 1,368 | | 1927 |  | 980 | 478 |  | 2,692 | 1,470 | | 5,040 | 3,875 | |
| *Daily Spray Operators Output* | | | | | | | | | | | | |  |
| Mean SOP daily target (structure /SOP) | | 18.0 | 25.0 |  | 18.0 | 25.0 |  | 18.0 | 25.0 | | 18.0 | 25.0 |  |
| Mean SOP daily output (structure /SOP) | | 19.6 | 26.3 |  | 8.7 | 25.0 |  | 11.3 | 21.4 | | 13.2 | 24.2 |  |
| Mean time spent spraying a room (mins) | | 7.1 | 2.2 |  | 5.8 | 2.7 |  | 5.6 | 3.5 | | 6.2 | 2.8 |  |
| Insecticide consumption Rooms/bottle | | 4.2 | 7.7 |  | 4.4 | 7.5 |  | 4.4 | 6.4 | | 4.3 | 7.2 |  |
| Average size of room (m^2^) | | 38.2 | 31.2 |  | 44.1 | 38.6 |  | 43.1 | 47.1 | | 41.8 | 39.0 |  |
| * SOP = Spray Operators  Mean daily man-hours for SOPs=6 hours (7:30am – 1:30pm) | | | | | | | | | | | | |  |

Supplementary Table S5: *Plasmodium falciparum* sporozoite infection rates and entomological inoculation rates (EIRs) in *An. gambiae* s.l. from full IRS, partial IRS, and unsprayed control communities, March 2019–December 2019

| Study Site | Total Tested | Number  Positive for *Pf* Sporozoites | Sporozoite  Rate | 95% Confidence Interval | | Mean  HBR | Mean  Monthly  EIR | Sum of  Monthly  EIRs* (10 months) |
| --- | --- | --- | --- | --- | --- | --- | --- | --- |
|  |  |  |  | **Lower**  **Limit** | **Upper**  **Limit** |  |  |  |
| BND |  |  |  |  |  |  |  |  |
| Kpemale (Full) | 306 | 7 | 2.3% | 0.6% | 4.0% | 1.8 | 1.51 | 15.1 |
| Bunbuna (Partial) | 601 | 3 | 0.5% | 0.1% | 1.1% | 4.7 | 0.58 | 5.8 |
|  |  |  |  |  |  |  |  |  |
| GUD |  |  |  |  |  |  |  |  |
| Banda-ya (Full) | 634 | 4 | 0.6% | 0.0% | 1.3% | 9.3 | 0.64 | 6.4 |
| Tum-Tu Zie (Partial) | 1,365 | 12 | 0.9% | 0.4% | 1.4% | 14.8 | 2.14 | 21.4 |
|  |  |  |  |  |  |  |  |  |
| KUD |  |  |  |  |  |  |  |  |
| Gbullung (Full) | 778 | 12 | 1.5% | 0.7% | 2.4% | 8.1 | 0.69 | 6.9 |
| Gupanarigu (Partial) | 657 | 4 | 0.6% | 0.0% | 1.2% | 9.5 | 1.05 | 10.5 |
|  |  |  |  |  |  |  |  |  |
| TML |  |  |  |  |  |  |  |  |
| Kulaa (Control) | 1,771 | 41 | 2.3% | 1.6% | 3.0% | 21.1 | 11.15 | 111.5 |
| Tugu (Control) | 1,383 | 33 | 2.4% | 1.6% | 3.2% | 18.4 | 10.59 | 105.9 |
| *Note: Pf=Plasmodium falciparum*  *^*^ Sum of monthly EIRs March*–December *2019.* | | | | | | | | |

Supplementary Table S6: Comparison of actual costs of a full IRS campaign and estimated costs of a partial IRS campaign in seven IRS districts in northern Ghana, 2018.

| Study Site | Actual Cost of 2018  30-day Full IRS | Estimated Cost of 2018 22.3-day  Partial IRS | Projected Savings |
| --- | --- | --- | --- |
| Daily Wages of Spray Team | |  |  |
| BYD | $23,965.37 | $17,814.26 | $6,151.11 |
| EMD | $28,999.43 | $21,556.24 | $7,443.19 |
| GUD | $20,345.68 | $15,123.62 | $5,222.06 |
| KAD | $17,951.00 | $13,343.58 | $4,607.42 |
| KUD | $16,235.15 | $12,068.13 | $4,167.02 |
| MMD | $9,317.44 | $6,925.97 | $2,391.48 |
| WMD | $16,392.88 | $12,185.38 | $4,207.51 |
| Sub-Total | $133,206.96 | $99,017.17 | $34,189.79 |
| Meals |  |  |  |
| BYD | $3,560.34 | $2,646.52 | $913.82 |
| EMD | $7,962.50 | $5,918.79 | $2,043.71 |
| GUD | $6,077.88 | $4,517.89 | $1,559.99 |
| KAD | $5,415.38 | $4,025.44 | $1,389.95 |
| KUD | $4,925.96 | $3,661.63 | $1,264.33 |
| MMD | $2,146.73 | $1,595.74 | $550.99 |
| WMD | $8,215.38 | $6,106.77 | $2,108.62 |
| Sub-Total | $38,304.19 | $28,472.78 | $9,831.41 |
| Transportation Related Cost (Vehicle Rental & Fuel) | |  |  |
| BYD | $22,236.95 | $16,529.47 | $5,707.48 |
| EMD | $21,639.14 | $16,085.10 | $5,554.05 |
| GUD | $15,998.48 | $11,892.21 | $4,106.28 |
| KAD | $18,834.27 | $14,000.14 | $4,834.13 |
| KUD | $15,718.62 | $11,684.18 | $4,034.45 |
| MMD | $13,582.23 | $10,096.12 | $3,486.11 |
| WMD | $32,055.95 | $23,828.25 | $8,227.69 |
| Sub-Total | $140,065.65 | $104,115.47 | $35,950.18 |
|  |  |  |  |
| Total Direct Spraying Cost | $311,576.80 | $231,605.42 | $79,971.38 |
|  | Percentage Savings | | 25.7% |

Supplementary Table S7: Estimated savings on insecticides under a partial IRS scenario based on a full IRS campaign conducted in seven districts in northern Ghana in 2018.

| District | Rooms Sprayed in 2018 | Full IRS in 2018 (actual) | |  | Partial IRS Scenario (estimated) | | | |
| --- | --- | --- | --- | --- | --- | --- | --- | --- |
|  |  | Insecticides Used (bottles) | Rooms sprayed/bottle (Full-spray) |  | Rooms sprayed/bottle (Partial-Spray) | Estimated Insecticides To be Used | Insecticide Savings if Partial-Spraying | *Estimated Savings ($) |
| BYD | 52,955 | 11,551 | 4.6 |  | 7.7 | 6,877 | 4,674 | $75,672.06 |
| EMD | 64,611 | 14,637 | 4.4 |  | 7.5 | 8,615 | 6,022 | $97,496.18 |
| GUD | 44,863 | 9,274 | 4.8 |  | 7.5 | 5,982 | 3,292 | $53,297.48 |
| KAD | 32,960 | 6,985 | 4.7 |  | 7.5 | 4,395 | 2,590 | $41,932.10 |
| KUD | 31,987 | 7,139 | 4.5 |  | 7.2 | 4,443 | 2,696 | $43,648.24 |
| MMD | 21,171 | 4,615 | 4.6 |  | 7.5 | 2,823 | 1,792 | $29,012.48 |
| WMD | 50,154 | 11,343 | 4.4 |  | 7.5 | 6,687 | 4,656 | $75,380.64 |
|  | 298,701 | 65,544 | 4.6 |  | 7.5 | 39,821 | 25,723 | $416,455.37 |
|  |  |  |  |  |  | Estimated % savings | | 39.2% |
| * using $16.19 price for Actellic CS, 30% ai, Syngenta for 2019 (shipping and insurance inclusive) | | | | | | | | |

**Supplementary File 2.**

**Modelling Analysis on the Impact of Partial Indoor Residual Spraying in Northern Ghana**

In 2018, experimental hut trials tested the impact of partial IRS of pirimiphos-methyl CS by deploying the insecticide to either: i) lower walls only; ii) upper walls only; iii) lower walls and ceilings; iv) upper walls and ceilings, or; v) fully to all walls and ceilings. One limitation of our predictions is that we fit simultaneously to the observed count data for *Anopheles* mosquitoes visiting the partially (or fully) sprayed huts without disaggregating by species. We do this due to the small sample size and absence of an understanding of the potential species specific effects of the IRS[^1^](#_ENREF_1).

**Characterising entomological impact from the experimental hut data**

Experimental hut data can be used to understand how sprayed insecticides can interfere with a mosquito feeding attempt and protect people from mosquito bites and thus malaria risk. The probable outcome of a mosquito feeding attempt indoors is assumed to end with either the mosquito successfully feeding, being repelled (deterred from outside or exiting without feeding from within the house) or being killed. The presence of an indoor residually sprayed (IRS) insecticide will alter this probability relative to a scenario with either no interventions or mosquito nets. This has been described by Le Menach (2007)[^2^](#_ENREF_2) and the effects incorporated into a transmission dynamics model to understand how this entomological effect translates to a public health impact[^3^](#_ENREF_3)^,^[^4^](#_ENREF_4). A systematic review of all experimental hut data (published up to 2017) has previously characterised the average impact of pirimiphos-methyl as the active ingredient in Actellic 300 CS^1^.

The studies included in this systematic review[^1^](#_ENREF_1) either provide no untreated nets or untreated nets with holes to the volunteer who sleeps within the hut to attract wild mosquitoes to feed. In the Ghanaian trials that we report, for ethical reasons, volunteers were protected with untreated and unholed mosquito nets. As such, the ultimate data represent a situation where a person is protected by both a net and an IRS application. In the transmission model, we already include the effects on insecticide-treated mosquito nets and assume that these are independent of the effects from IRS. Therefore, we fit statistical models to the mortality and deterrence data observed in Ghana – to capture the different impacts of the partial spray campaign – and then use the proportional blood-feeding and exiting data collated in the systematic review to provide ‘IRS only’ parameter sets for the modelling exercise described below.

The experimental hut data collated in 2018 in Ghana is used to estimate the variable impact that partial IRS of walls and ceilings inside houses may have. Briefly, a flexible logistic function is fitted to the pirimiphos-methyl experimental hut data (fitting to Ghana trial data for Equations 1, 2, 5 and 6; systematic review data[^1^](#_ENREF_1) for Equations 3 and 4) to capture changing impact on probable outcomes of mosquito feeding attempts over time (*t*, in days since the application of the IRS),

$l_{S}=\frac{1}{1+\exp\left( -\left( l_{S\vartheta}+l_{S\gamma} \times t \right) \right)}$ (1)

$N_{dead}\sim binomial\left( l_{s}, N_{total1} \right)$ (2)

$k_{S}=\frac{k_{0}}{1+\exp\left( -\left( k_{S\vartheta}{+k}_{S\gamma} \times t \right) \right)}$ (3)

$N_{successfully\_fed}\sim binomial\left( k_{s}, N_{total1} \right)$ (4)

$m_{S}=\frac{1}{1+\exp\left( -\left( m_{S\vartheta}{+l}_{S\gamma} \times t \right) \right)}$ (5)

$N_{deterred}\sim binomial\left( m_{s}, N_{total2} \right)$ (6)

The proportion of mosquitoes being killed $l_{S}$ over time depends on the initial efficacy ($l_{S\vartheta}$) and the duration of the impact ($l_{S\gamma}$). A logistic binomial model is fitted to the total number of mosquitoes killed (*N_dead_*) in the sprayed huts (*N_total1_*). The proportion of mosquitoes successfully feeding ($k_{S}$) and being deterred away from a sprayed hut ($m_{S}$) are determined similarly (Equations 3 - 6). Deterred mosquitoes (*N_deterred_*) are calculated from the difference between mosquitoes in control and sprayed huts (summed total, *N_total2_*). Given the uncertainty in the measurement for deterrence (a comparison between the total number of mosquitoes entering the control and treated hut over time), we assume the deterrence effect decays with the mortality effect (Table S8). Whether a mosquito is deterred from the sprayed household (*m_S_*) determines the probabilities that a mosquito will be killed (*l_s_*) or successfully blood-fed (*k_s_*). The proportion of mosquitoes that enter and are then repelled without being killed or feeding is then *j_s_ = 1 – l_s_ – k_s_*. An estimated parameter *k_0_*  (0.699[^5^](#_ENREF_5)^,^[^6^](#_ENREF_6)) represents the proportion of mosquitoes successfully feeding in the absence of any indoor interventions. The functions *k_s,_ l_s_* and *j_s_* ­­(the mosquito is neither blood-fed or killed) are adjusted by the degree of deterrence (*m_s_*, which is now a time varying quantity) measured in the experimental huts as follows,

${l'}_{S}=l_{S}\times\left( 1-m_{S} \right)$ (7)

${k'}_{S}=k_{S}\times\left( 1-m_{S} \right)$ (8)

${j'}_{S}=$ $j_{S}\times\left( 1-m_{S} \right)+m_{S}$. (9)

The per feeding attempt probability that a mosquito entering a sprayed hut successfully blood-feeds, exits without feeding or dies, denoted $s_{S}$, $r_{S}$ and $d_{S}$ respectively, are:

$s_{s}=\frac{{k'}_{S}}{k_{0}}$ (10)

$r_{s}=\left( 1-\frac{{k'}_{S}}{k_{0}} \right)\times\left( \frac{{j'}_{S}}{{l'}_{S}+{j'}_{S}} \right)$ (11)

$d_{s}=\left( 1-\frac{{k'}_{S}}{k_{0}} \right)\times\left( \frac{{l'}_{S}}{{l'}_{S}+{j'}_{S}} \right)$. (12)

This statistical comparison yields parameter estimates (Table S8) that can be employed in a transmission dynamics model for *Plasmodium falciparum* malaria that mechanistically captures the passage of malaria between people and mosquitoes at the population level[^3^](#_ENREF_3)^,^[^4^](#_ENREF_4).

**Supplementary Table S8: Parameters reflecting the distinct entomological impact of partial IRS of pirimiphos-methyl derived from the experimental hut studies in Ghana or systematic review**[**^1^**](#_ENREF_1)**.** Mean (and 90% CrI) parameter estimates for Equations 1 – 6. Parameters determine the time dependent impacts of partial IRS. These are distinct given the results of the experimental hut survey. We show in the final column the default pirimiphos-methyl determined from the systematic review[^1^](#_ENREF_1). These parameters are combined into a transmission dynamics model for Plasmodium falciparum ^7^ which can be used to theoretically explore epidemiological impact.

|  | Spray strategy investigated in the experimental huts | | | | |
| --- | --- | --- | --- | --- | --- |
| Parameter | Lower walls only | Upper walls only | Lower walls and ceilings | Upper walls and ceilings | Fully sprayed hut |
| $l_{S\vartheta}$ | -0.265  (-0.700 – 0.164) | 0.292  (-0.175 – 0.846) | 0.969  (0.569 – 1.436) | 0.983  (0.610 – 1.399) | 1.916  (1.468 – 2.402) |
| $l_{S\gamma}$ | -0.017  (-0.024 – 0.009) | -0.031  (-0.043 – -0.021) | -0.026  (-0.035 – -0.017) | -0.028  (-0.036 – -0.020) | -0.037  (-0.047 – -0.028) |
| $k_{S\vartheta}$ | -2.222  (-2.292 – -2.162) | -2.222  (-2.292 – -2.165) | -2.222  (-2.284 – -2.139) | -2.222  (-2.291 – -2.156) | -2.222  (-2.285 – -2.156) |
| $k_{S\gamma}$ | 0.008  (0.008 – 0.009) | 0.008  (0.008 – 0.009) | 0.008  (0.008 – 0.009) | 0.008  (0.008 – 0.009) | 0.008  (0.008 – 0.009) |
| $m_{S\vartheta}$ | -0.153  (-0.369 – 0.061) | 0.574  (0.322 – 0.775) | -0.243  (-0.415 – -0.020 | -0.160  (-0.373 – 0.095) | -0.295  (-0.481 – -0.106) |
| $m_{S\gamma}$ | -0.017  (-0.025 – 0.009) | -0.031  (-0.043 – -0.024) | -0.026  (-0.035 – -0.018) | -0.028  (-0.036 – -0.021) | -0.037  (-0.047 – -0.030) |

The estimates above feed into the transmission model by adjusting the probability of a mosquito feeding attempt resulting in a successful bite (*w_i_*) on an individual *i*, being bitten at all (*y*_i_), or being repelled (*z_i_*) (Table S9) as has been extensively described in Griffin et al (2010)^3^ and (2016)^7^ . These estimates are adjusted by the proportion of mosquitoes feeding indoors (*φ_I_*) or in bed (*φ_B_*) in the absence of an indoor intervention.

**Supplementary Table S9 . Probabilities of successful feeding, biting and repulsion for combinations of LLIN/IRS interventions**

|  | IRS only | LLINs only | IRS plus LLINs |
| --- | --- | --- | --- |
| Probability of successful feeding (*w_i_*) | $1-\Phi_{I}+\Phi_{I}(1-r_{S})s{}_{S}$ | $1-\Phi_{B}+\Phi_{B}s_{N}$ | $\begin{matrix} 1-\Phi_{I}+\Phi_{ B}(1-r_{S})s_{N}s_{S} \\ +(\Phi_{I}-\Phi_{B})(1-r_{S})s_{S} \end{matrix}$ |
| Probability of biting (*y_i_*) | $1-\Phi_{I}+\Phi_{I}(1-r_{S})$ | $1-\Phi_{B}+\Phi_{B}s_{N}$ | $\begin{matrix} 1-\Phi_{I}+\Phi_{B}(1-r_{S})s_{N} \\ +(\Phi_{I}-\Phi_{B})(1-r_{S}) \end{matrix}$ |
| Probability of repellency (*z_i_*) | $\Phi_{I}r_{S}$ | $\Phi_{B}r{}_{N}$ | $\Phi_{B}(1-r_{S})r_{N}+\Phi_{I}r_{S}$ |

The respective probable outcomes elicited from partially spraying the indoor areas of the experimental huts in Ghana are compared to estimate potential variability in the entomological impact (Figure 3 in the main manuscript). In Figure S7 panel 1, we first show the observed *Anopheles* mosquito mortality data in each partial spray strategy (as Figure 3, main manuscript) before demonstrating the average probable outcome of a mosquito feeding attempt over time; the mosquito may be killed (blue) or deterred (green) as determined from the Ghanaian experimental hut data. However, in each case we assume the same probable feeding success (red) in the presence of only IRS as determined using the observed data collated in the systematic review[^1^](#_ENREF_1). A probable outcome of mosquitoes neither feeding nor being killed is adjusted accordingly and shown in yellow.


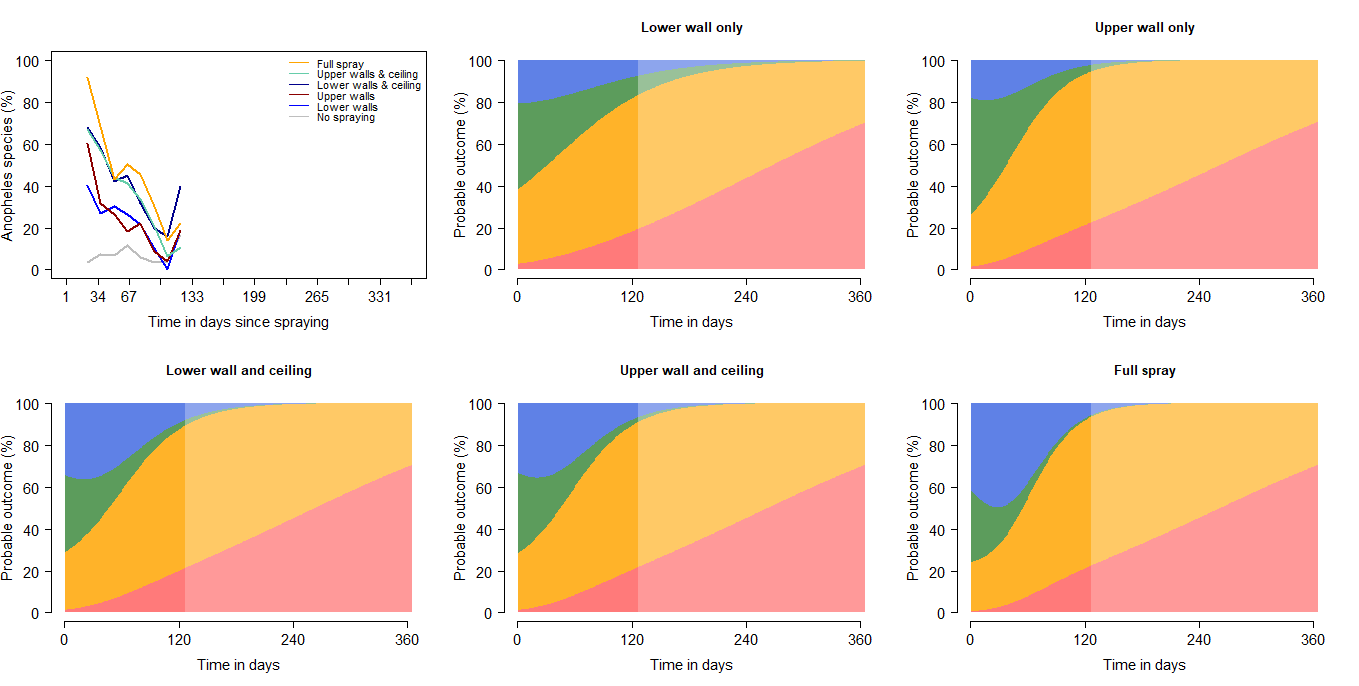


**Supplementary Figure S7. The probable outcome of a mosquito feeding attempt given full or partially sprayed walls and ceilings.** Pirimiphos-methyl insecticide was sprayed onto either: i) the lower walls only; ii) the upper walls only; iii) the lower walls and ceilings; iv) the upper walls and ceilings, or; v) fully to all walls and ceilings as noted on the panel headings. The first panel shows the observed mortality data for the partial spray arms measured in the Ghanaian experimental hut trial over 126 days (18 weeks of observations). In each subsequent panel, the average probability of a mosquito being killed (blue), deterred (green), exiting without feeding (orange) or successfully blood feeding (red) are shown as estimated using the experimental hut data on mortality and deterrence from Ghana and the blood-feeding data observed from the systematic review[^1^](#_ENREF_1). Darker regions represent the statistical fits to data given the observations from the Ghanaian trial and lighter sections indicate the days of the year for which we are inferring given the statistical model fits.

**The potential epidemiological effect of partial IRS**

Table S10 provides the data resources to inform the transmission model for a location like Northern Ghana. The transmission model that we use tracks transmission of *Plasmodium falciparum* between human hosts and *Anopheles* mosquito vectors. The differential equations and associated assumptions of the original transmission model [^3^](#_ENREF_3) have been comprehensively reported in the Supplementary Material from Griffin et al.[^4^](#_ENREF_4), Walker et al. [^7^](#_ENREF_7)and Winskill et al.[^8^](#_ENREF_8). The model has been extensively fitted to data on the relationship between vector density, EIR, parasite prevalence, uncomplicated malaria, severe disease and death[^3^](#_ENREF_3)^,^[^4^](#_ENREF_4)^,^[^9-11^](#_ENREF_9). Unless stated in Tables S8 & S11, default parameters are taken from these papers. The code is published and freely available here: [https://github.com/jamiegriffin/Malaria_simulation](https://eur01.safelinks.protection.outlook.com/?url=https%3A%2F%2Fgithub.com%2Fjamiegriffin%2FMalaria_simulation&data=02%7C01%7Cj.griffin%40qmul.ac.uk%7C8748d595ecb9480178e608d7104dc53c%7C569df091b01340e386eebd9cb9e25814%7C0%7C0%7C636995798526779900&sdata=eAatqwDkTiYmBIkQq1yVpbL6ewsyS%2FjEBkGJ%2FYW1Bmw%3D&reserved=0).

**Supplementary Table S10: Data values used for model parameters:** A description of the available information and data for Northern Ghana to inform the transmission model

| Parameter / Data | Description | Resource | Mean (Range) used |
| --- | --- | --- | --- |
| ITN_frequency | Regularity of spray campaign | MIS / DHS / personal communication PMI/Abt | 1, annual; Top up every year through routine campaigns |
| ITN_cover | Percentage of people sleeping under nets at the start of the net campaign (usage is modelled to wane over time) | School-based distribution occurs annually in May + ANC top-up. *Surveys conducted in 2017/2018 suggest that 38 – 57% of people were using nets in the village trial spray regions[^12^](#_ENREF_12) | 48% |
| ITN cover in the years preceding mass campaign | Net cover in 2016-2017 and previous | The last time ITNs were distributed in the Northern Region through a mass campaign was 17^th^ to 18^th^ April, 2016. Since then, IRS districts have not received nets through mass campaigns. | 48% |
| Year and month of net campaign | Timing of last mass net campaign | May 2018 generally except in pilot spray regions (school-based top up in 2019) | May 2019 |
| ITN efficacy parameters determined by levels of pyrethroid resistance in local mosquito | Background level of pyrethroid resistance in the local Anopheles genus, percentage survival in discriminatory dose bioassay | Abuaku et al 2018; personal communication; efficacy determined using Churcher et al 2016 | 40 – 80% pyrethroid resistance (proportion surviving the bioassay test) |
| IRS_cover | Percentage of people in houses sprayed with insecticide | Manuscript in prep (Ghana data) | 90% |
| Year and month for IRS | Timing of IRS campaign | Manuscript in prep (Ghana data) | 16^th^ May 2019 |
| ACT treatment | Percentage of people receiving ACT as treatment for clinical malaria | We assume 40% of people receive clinical treatment in Northern Ghana, we ignore malaria in pregnancy treatment (which is high in Ghana), WHO Malaria reports | 67% of 40% |
| Non-ACT treatment | Percentage of all people receiving treatment if known (total – ACT gives percentage non-ACT treatment) |  | 33% of 40% |
| Prevalence in children under 5 years at baseline | Ideally, month and year of survey to estimate prevalence in U5s | DHS/MIS/Malaria Atlas Project | 18.7% MIS 2019 |
| Mosquito species | Proportion of mosquito species present | Manuscript in prep (Ghana data) | 96% *An. gambiae*  4% *An. arabiensis* |
| ITN_IRS_corr | Correlation between people receiving nets and spray | Assumed | 1 (those with nets also receive spray) |
| SMC cover | Above 80% in all districts in Northern Ghana (Karaga 84.23%; Gushegu 89.86%; Kumbungu 85%; Tatale-Sangule 89.07%) | | |
| Reps | 4 rounds | | |
| SMC start | 20 July 2019 | | |
| Age cohort for SMC | 3-59 months | | |
| Year of initiation | In Northern Region (now split into Northern, North East, and Savannah Regions), SMC started in 2019 | | |
| District | To match seasonal patterns in mosquito densities we use default parameters that are matched to Northern Ghana[^7^](#_ENREF_7) | | |
| Population in Northern Ghana | 2019 = 1,997,946; 2020 = 2,055,886 | | |

Table S11: Parameters that are specified for the simulations of Northern Ghana. These are kept constant across the partially sprayed IRS scenarios and are informed by the information in Table S10.

| Parameter | Definition |  |
| --- | --- | --- |
| *d*_N0_ | Probable mortality of a mosquito feeding attempt due to a pyrethroid mosquito net working optimally immediately after distribution | 0.51 (0.50 – 0.52) |
| *r*_N0_ | Probable repellence effect from a mosquito feeding attempt due to a pyrethroid mosquito net working optimally immediately after distribution | 0.30 (0.30 – 0.31) |
| Λ | Half-life of insecticidal effect of a pyrethroid mosquito net (years) | 1.98 (1.50 – 2.42) |
| *M* | Ratio of mosquitoes to people in the model required to match observed prevalence estimates (see Table MS3) | 7.00 (6.13 – 8.24) |
| *Q*_0_ | Anthropophily, proportion of mosquito bites on people | *An. gambiae* s.l. 92%  *An. arabiensis* 71% |
| *φ*_I_ | Proportion of mosquito bites received indoors in the absence of vector control interventions | *An. gambiae* s.l. 90%  *An. arabiensis* 86% |
| *φ_B_* | Proportion of mosquito bites received in bed in the absence of vector control interventions | *An. gambiae* s.l. 85%  *An. arabiensis* 80% |

**Predicting the epidemiological impact of partially IRS pirimiphos methyl**

A generic scenario that broadly reflects the Northern region of Ghana is used to explore the potential lost protective efficacy (measured as the cases averted per 1,000 people per year, and the relative reduction in prevalence after 2, 4, 6 and 8 months since spraying that is provided by the respective spray campaign strategy, Table 1, main manuscript) that occurs when IRS is administered to the different sections of the wall or ceilings measured in the experimental huts. Ghana is generally a perennial transmission setting but in this northern region, there is a peak in transmission in June / July hence the spray campaign takes place in May, at the optimal time relative to this transmission peak.

We make simplifying assumptions to focus our inference from the modelling exercise on the altered impact of IRS when partially sprayed. First, we assume that there is no change in the level of pyrethroid resistance in Northern Ghana throughout the period we are simulating. The estimated pyrethroid resistance changes the effectiveness of pyrethroid insecticide treated mosquito nets. This is estimated by the proportion of mosquitoes surviving exposure to a discriminatory dose of pyrethroid insecticide in a standardized susceptibility bioassay test[^13^](#_ENREF_13). In the most recent surveys this estimate is highly variable ranging between 10% and 90% depending on species and site (personal communication, J Armistead). Therefore, we simulate a range of background levels of pyrethroid resistance in the local *Anopheles* genus which corresponds to 40 – 80% of mosquitoes surviving in the discriminatory dose bioassay[^14^](#_ENREF_14) and acts to reduce the efficacy of mosquito nets.

Net use in Northern Ghana is recorded as 58.2% (Malaria Indicator Survey 2019) but in regions where IRS is used, the last mass campaign was 2014-2015. In spray areas, a school-based top up campaign happens annually (the latest in May 2019). All children of school grade 2 and 6 received nets. Surveys completed in 2017/2018 suggest that 38 – 57% of people were using nets around the time of the pilot study in spray regions[^12^](#_ENREF_12). Therefore, we assume ‘top-up’ of mosquito nets from both school grade and alternative sources such as anti-natal clinics result in a continuous mosquito net usage of 48% (total) for the simulations. Gradually both insecticide efficacy and adherence to use are modelled to wane across the next 3-years following a new net being deployed, which is reflected in reduced protection from pyrethroid mosquito nets.

The other key preventative intervention that is used in Northern Ghana is seasonal malaria chemoprevention (SMC). In 2019, SMC was introduced across Northern Ghana as a presumptive, prophylactic treatment for children aged 3 to 59 months that is delivered monthly for 4 months starting 20^th^ July 2019. The intervention covered more than 80% of children in each district measured. The high SMC cover will reduce the observed impact from IRS, particularly within the targeted age cohort, but this will be consistent between the full and partial IRS strategies. SMC makes a bigger difference to reduce malaria in the target age cohort (children 3 – 59 months of age), than other age groups.

Given the high coverage of rooms sprayed in the pilot study we simulate that 90% of people are protected by the IRS application (IRS application is simulated to take place simultaneously on May 16^th^, 2019). Different effectiveness of the IRS intervention is then driven by the efficacies estimated using the experimental hut data for the respective spray scenarios (lower walls, upper walls, lower walls and ceilings, upper walls and ceilings, or full cover) (Figure S7). We assume no resistance to pirimiphos methyl, the active ingredient in pirimiphos methyl[^14^](#_ENREF_14).

**Limitations and assumptions**

We assume that 40% of clinically ill patients receive treatment, of which 67% is artemisinin combination therapy (ACT). From the modelling perspective this provides prolonged prophylaxis for the patient relative to the non-ACT treatment which makes up the remaining case management assumed. We further assume this does not change throughout the simulations. We assume that no other interventions are operating in this simulation. Ghana has achieved a very high rate of preventative treatment for pregnant women (78%) (PMI, Ghana profile) but we ignore this intervention for simplicity as it would not relevantly change the simulated IRS impact given that the impact from case management of pregnant women would be consistent across all trial arms.

To ensure that we both capture the seasonality of malaria transmission and the relative burden of the Northern region of Ghana, the model is calibrated to match estimates of prevalence. PCR prevalence in children aged 6-months to 5 years from the Malaria Indicator Survey 2019 is estimated to be 18.7% as measured in Sept-Nov 2019. However, this is after the net, SMC and IRS deployment of the trial year. The model is calibrated to estimate slide prevalence in children aged 2 to 10 years from the Malaria Atlas Project (MAP) in 2017 (24.7%) and we confirm that the 18.7% PCR prevalence in 6 to 59-month year old children is representative (Figure S8). In the model, transmission is driven by the entomological inoculation rate (EIR), the number of infectious mosquito bites received per person each year. To match both the seasonality experienced in Northern Ghana and to recover the level of malaria burden observed, we arbitrarily increase the ratio of mosquitoes to humans until the baseline burden of malaria matches that observed (24.7% slide prevalence in children 2 to 10 years of age in 2017). The average seasonality of Northern Ghana is estimated given 8-years of rainfall data (2003 - 2010)[^15^](#_ENREF_15)^,^[^16^](#_ENREF_16), a 1-month lag is introduced to allow mosquito yearly-average peak abundance to be recovered.

***
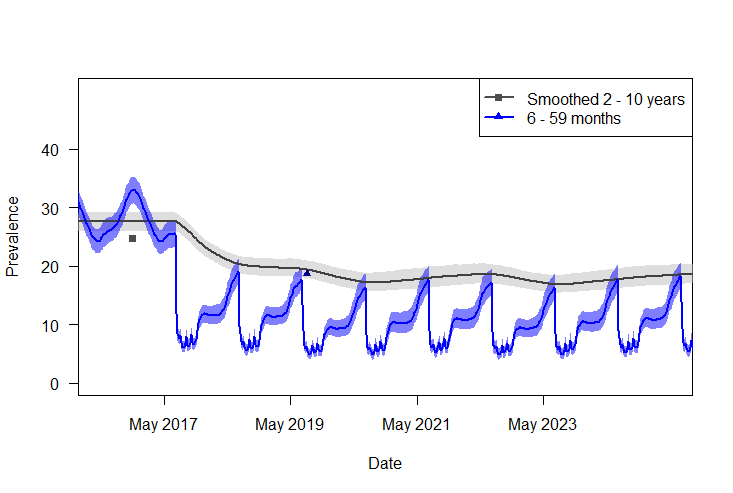
***

**Supplementary Figure S8**: **Calibrating the model to the 2 estimates of prevalence available during the trial period for Northern Ghana.** The grey line indicates the smoothed prevalence across years for 2 – 10-year old children, which is used to match to the Malaria Atlas Project to projections for 2017 (24.7%). The blue line indicates the corresponding prevalence, by week, in children 6 to 59 months of age which matches to the Malaria Indicator Survey 2019 where estimates were made between September and November. The shaded polygons indicate the model uncertainty (95% intervals). Seasonal malaria chemoprevention begins in July 2019 and can be seen in the predicted weekly prevalence for children 6 to 59 months of age during July – August each year.

Further simplifying assumptions include the use of parameter estimates to summarize the bionomics of mosquito species that remain constant throughout the year, so the model does not account for any potential changes in behavior with temperature or season other than density. Experimental hut data are also variable due to differences in the size of the huts, wall surfaces, baffle size, shape and location, hut wall and ceiling surface and the intervention provided for the control hut sleeper (e.g. no net, a holed untreated net)[^17^](#_ENREF_17) We parameterize the IRS (and mosquito net[^13^](#_ENREF_13) effects) on generic mosquitoes in the absence of sufficient data to power analyses that can distinguish effects between species. To mitigate for this, we include uncertainty in the model projections for intervention efficacy estimates. From the uncertainty bounds measured in the Bayesian statistical analysis of the experimental hut data we generate 1000 posterior predictive parameter estimates for the model simulations.

**Comparable effectiveness with full and partial IRS**

As noted in the main manuscript, the transmission model simulations provide scenarios to compare the potential public health impact of partially spraying pirimiphos-methyl IRS. We simulate the all-age and under 5-year old clinical cases per 1,000 people per year for 1-year following the spray campaigns for each of the simulation scenarios outlined in Table 1 in the main manuscript. We also provide a comparison of prevalence at 2, 4, 6 and 8-months after spraying (Table 1 in the main manuscript). These metrics provide measures to compare effectiveness between a full spray and various partial spray scenarios relative to no IRS campaign. To do this, we use the efficacy equation:

$$\% Efficacy=100 \times\frac{\left( C-T \right)}{C}$$

Where *C* is the counterfactual trial arm without any IRS deployed and *T* is the trial arm with some level of IRS implemented be it partial or fully sprayed.

These estimates are used to generate cost-effectiveness estimates for the application of partial spraying where only upper walls and ceilings are covered. To do this, we estimate the all-age clinical cases averted per strategy (partial: upper walls and ceiling; full: full spray) per person per year following the IRS campaign. We multiply this by the cost per person for the partial or full spray strategy tested in the pilot to give the cost per case averted.

**References**

1 Sherrard-Smith, E. *et al.* Systematic review of indoor residual spray efficacy and effectiveness against Plasmodium falciparum in Africa. *Nature communications* **9**, 1-13 (2018).

2 Le Menach, A. *et al.* An elaborated feeding cycle model for reductions in vectorial capacity of night-biting mosquitoes by insecticide-treated nets. *Malaria journal* **6**, 1-12 (2007).

3 Griffin, J. T. *et al.* Reducing Plasmodium falciparum malaria transmission in Africa: a model-based evaluation of intervention strategies. *PLoS Med* **7**, e1000324 (2010).

4 Griffin, J. T. *et al.* Potential for reduction of burden and local elimination of malaria by reducing Plasmodium falciparum malaria transmission: a mathematical modelling study. *The Lancet Infectious Diseases* **16**, 465-472 (2016).

5 Curtis, C., Myamba, J. & Wilkes, T. Comparison of different insecticides and fabrics for anti‐mosquito bednets and curtains. *Medical and veterinary entomology* **10**, 1-11 (1996).

6 Lines, J., Myamba, J. & Curtis, C. Experimental hut trials of permethrin‐impregnated mosquito nets and eave curtains against malaria vectors in Tanzania. *Medical and veterinary entomology* **1**, 37-51 (1987).

7 Walker, P. G., Griffin, J. T., Ferguson, N. M. & Ghani, A. C. Estimating the most efficient allocation of interventions to achieve reductions in Plasmodium falciparum malaria burden and transmission in Africa: a modelling study. *The Lancet Global Health* **4**, e474-e484 (2016).

8 Winskill, P., Slater, H. C., Griffin, J. T., Ghani, A. C. & Walker, P. G. The US President's Malaria Initiative, Plasmodium falciparum transmission and mortality: A modelling study. *PLoS medicine* **14**, e1002448 (2017).

9 Griffin, J. T., Ferguson, N. M. & Ghani, A. C. Estimates of the changing age-burden of Plasmodium falciparum malaria disease in sub-Saharan Africa. *Nature communications* **5**, 1-10 (2014).

10 White, M. T. *et al.* Modelling the impact of vector control interventions on Anopheles gambiae population dynamics. *Parasites & vectors* **4**, 153 (2011).

11 Griffin, J. T. *et al.* Gradual acquisition of immunity to severe malaria with increasing exposure. *Proceedings of the Royal Society B: Biological Sciences* **282**, 20142657 (2015).

12 PMI/VL. The PMI Vectorlink Project. Ghana Operational Research (Effect of Indoor Residual Spraying on Anopheles vector behaviors and their impact on malaria transmission in northern region of Ghana ) Final Report (2019).

13 Churcher, T. S., Lissenden, N., Griffin, J. T., Worrall, E. & Ranson, H. The impact of pyrethroid resistance on the efficacy and effectiveness of bednets for malaria control in Africa. *Elife* **5**, e16090 (2016).

14 Abuaku, B. *et al.* Impact of indoor residual spraying on malaria parasitaemia in the Bunkpurugu-Yunyoo District in northern Ghana. *Parasites & vectors* **11**, 555 (2018).

15 Garske, T., Ferguson, N. M. & Ghani, A. C. Estimating air temperature and its influence on malaria transmission across Africa. *PloS one* **8**, e56487 (2013).

16 National Weather Service, N. W. *Climate Prediction Center*, <<https://www.cpc.ncep.noaa.gov/>> *[cited 24 Mar 2016]*.

17 Massue, D. J. *et al.* Comparative performance of three experimental hut designs for measuring malaria vector responses to insecticides in Tanzania. *Malaria journal* **15**, 1-11 (2016).
